# Cation Exchange Membranes and Process Optimizations in Electrodialysis for Selective Metal Separation: A Review

**DOI:** 10.3390/membranes13060566

**Published:** 2023-05-30

**Authors:** Önder Tekinalp, Pauline Zimmermann, Steven Holdcroft, Odne Stokke Burheim, Liyuan Deng

**Affiliations:** 1 Department of Chemical Engineering, Norwegian University of Science and Technology (NTNU), 7491 Trondheim, Norway; onder.tekinalp@ntnu.no; 2 Department of Energy and Process Engineering, Norwegian University of Science and Technology (NTNU), 7491 Trondheim, Norway; pauline.zimmermann@ntnu.no (P.Z.); odne.s.burheim@ntnu.no (O.S.B.); 3 Department of Chemistry, Simon Fraser University, Burnaby, BC V5A 1S6, Canada; holdcrof@sfu.ca

**Keywords:** monovalent selective cation exchange membranes, electrodialysis, metal recovery, ion selectivity, membrane properties, ionic characteristics, boundary layer, membrane preparation, process conditions, current density

## Abstract

The selective separation of metal species from various sources is highly desirable in applications such as hydrometallurgy, water treatment, and energy production but also challenging. Monovalent cation exchange membranes (CEMs) show a great potential to selectively separate one metal ion over others of the same or different valences from various effluents in electrodialysis. Selectivity among metal cations is influenced by both the inherent properties of membranes and the design and operating conditions of the electrodialysis process. The research progress and recent advances in membrane development and the implication of the electrodialysis systems on counter-ion selectivity are extensively reviewed in this work, focusing on both structure–property relationships of CEM materials and influences of process conditions and mass transport characteristics of target ions. Key membrane properties, such as charge density, water uptake, and polymer morphology, and strategies for enhancing ion selectivity are discussed. The implications of the boundary layer at the membrane surface are elucidated, where differences in the mass transport of ions at interfaces can be exploited to manipulate the transport ratio of competing counter-ions. Based on the progress, possible future R&D directions are also proposed.

## 1. Introduction

The use of metal species has considerably increased with the progress of the industrialized world, leading to the rapid depletion of resources and hazardous waste streams discharged into the surface water [1]. Typically, metal species are present in various sources, such as spent acids or electroplating processes that mainly involve transition metal ions, which are generally known as industrial metals (i.e., Ag^+^, Cu^2+^, Ni^2+^, and Fe^3+^) and seawater having a high amount of alkali (i.e., Li^+^, Na^+^, and K^+^) or alkaline earth metals (i.e., Mg^2+^ and Ca^2+^) [2,3]. Even the treated streams of these sources contain metal ions in various amounts and compositions. Given the significance of the wide use of these metal species in diverse industries, their selective separation is imperative to recover metal values, conserve natural metal resources, and avoid environmental pollution. Traditional technologies (i.e., adsorption, precipitation, and extraction) have been applied successfully to selectively recover metal ions. However, there still exist serious drawbacks, such as the use of highly hazardous and toxic chemicals, the production of large amounts of sludge, incomplete metal removal, and metal precipitation [4]. Given the stringent environmental standards, waste production should be minimized, and high recovery of targeted metal ions must be achieved.

Cation exchange membranes (CEMs), on the other hand, are separation membranes that continue attracting considerable attention to treat metal-contaminated water, particularly in technologies involving electrodialysis [5]. Electrodialysis can offer numerous advantages compared to conventional technologies, such as excellent selective recovery, treatment of dilute concentrations of targeted ions, and a more environmentally friendly operation [6,7]. Unlike other membrane processes that primarily focus on water purification, electrodialysis targets more mineral recovery.

CEMs can be considered cation exchange polymers and resins in the film form [8], usually carrying pendant sulfonic groups in the polymer matrix that separate cations in a solution of opposing charge (counter-ions) from anions having the same ionic charge as the fixed ionic groups (co-ions). However, when the preferential permeation of multiple metal counter-ions of different or equal valency in solution is desirable, it is not ideal with standard CEMs, thus restricting many potential industrial applications of CEMs. Hence, strategies to enhance the selectivity of one metal ion over another one of the same or different valences in a mixture are highly desirable. One strategy is to design selective CEMs for a particular metal ion, and another strategy is to control operating conditions to favor the transport of a targeted metal ion over others. Understanding the characteristics of target ions, the membrane properties, and transport basics are paramount to achieving the intended selectivity.

Several review articles have addressed ion selectivity of counter-ions in the literature. Sata et al. [9] reviewed the modification of CEMs for selectivity among cations in 2003. In 2017, Xu and co-workers [10] summarized monovalent cation selective membranes with an emphasis on fabrication strategies for diffusion dialysis and electrodialysis applications, while Khoiruddin et al. [11] described approaches to surface modification of IEMs and monovalent ion selectivity. Luo et al. [12] published a comprehensive review of IEMs in 2018, including the separation of both anions and cations, focusing on advancements in fabrication methods, ion transport mechanisms, and experimental methods to determine ion selectivity. More recently, a review of various types of membrane processes for alkali and alkaline-earth metal ions separation was provided by Wang et al. [13], in which monovalent selective CEMs in electrodialysis were included as a section. Juve et al. [4] also discussed the types, limitations, and challenges of the electrodialysis process, but mostly for the removal of metal ions from acid effluents, with only a small section for the selective separation of metal ions. 

In the current study, a comprehensive review of the research progress and recent advances of CEMs for enhanced monovalent cation selectivity are presented. Both the membrane material development and process optimization aspects are discussed, including CEMs’ surface modification, membrane matrix regulation, incorporation of additives as well as solution/membrane boundary layers, mass transport characteristics in the solution, and the influence of the operating conditions on metal ion selectivity. For the reader’s convenience, we have briefly outlined relevant theory and background, including the ion selectivity concept and the characteristics of selected metal cations (i.e., hydrated radius, valency, and hydration energy) to explain ionic permeation ability through CEMs. The structure–property relationships of CEMs are discussed to connect membrane properties to ion selectivity, focusing on the key membrane properties that play key roles in ion sorption and mobility, i.e., membrane charge, water uptake, and polymer morphology. Transport phenomena affecting the ion fluxes and CEM selectivity mechanisms in the electrodialysis process design are also discussed. The implications of ion transport across the boundary layer and its role in counter-ion selectivity and the formation of a boundary layer are analyzed for cation selectivity enhancement. Progress in membrane design, the effect of stack configuration/design and solution properties, and operating conditions on metal ion selectivity are elaborated. Lastly, strengths, challenges, and possible future research and development directions on monovalent selective CEMs are proposed.

## 2. Theory and Background

### 2.1. Electrodialysis Process

**Figure 1:**
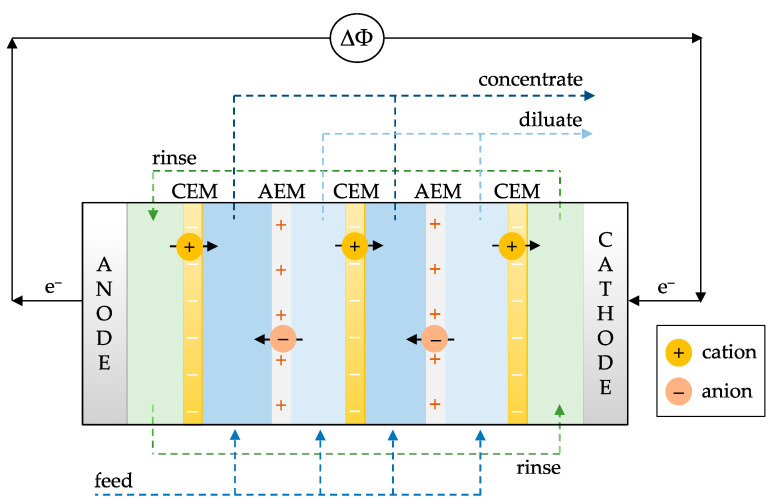
Illustration of an electrodialysis stack. CEMs and AEMs are alternated between the electrodes. A rinse solution is circulated in the electrode compartments, where electrochemical reactions take place [7].

Electrodialysis is an ion-exchange membrane (IEM) process for ion separation or accumulation by applying an electric current across the cell [14]. In an electrodialysis process, ions transport from one compartment to another through IEMs under the applied potential as a driving force. Figure 1 illustrates a typical electrodialysis cell consisting of several cell pairs (an anion exchange membrane (AEM)), a dilute compartment, a CEM, and a concentrate compartment) sandwiched between an anode and a cathode. Since electrochemical reactions take place at the electrodes that deplete or create ions, causing a charge imbalance and the formation of an electric field across the cell. As a result, cations migrate toward the cathode, passing through the CEM, and reciprocally, anions migrate toward the anode through the AEM. Consequently, each second compartment is desalinated while ions accumulate in each alternate compartment.

### 2.2. Ion Selectivity

Ion selectivity $\left[P_{B}^{A}\right]$ is often defined to be the relative rate of permeance of the desired counter-ions to the additional feed counter-ions. $P_{B}^{A}>$ 1 indicates a favorable transport of component A with respect to component B. The ion permeance ratio is related to the ionic fluxes and their feed concentrations. The selectivity of two counter-ions can be calculated as follows [9]:

(1) $$P_{B}^{A}=\frac{t_{A}C_{B}}{t_{B}C_{A}}$$

where C_A_ and C_B_ (mol⋅L^−1^) are the concentrations at the membrane surface of the desalting side of the system, and t_A_ and t_B_ (mol⋅m^−2^⋅s^−1^) are the transport number of the components A and B. 

The separation efficiency, S(t) (%), is also used to parameterize the selective separation of components A and B and is expressed as follows [15]:

(2) $$S\left(t\right)\left(\%\right)=\frac{\left[\frac{C_{B}\left(t\right)}{C_{B}\left(0\right)}\right]-\left[\frac{C_{A}\left(t\right)}{C_{A}\left(0\right)}\right]}{\left[1-\frac{C_{B}\left(t\right)}{C_{B}\left(0\right)}\right]+\left[1-\frac{C_{A}\left(t\right)}{C_{A}\left(0\right)}\right]}\cdot 100\%$$

where C_A_(0) and C_B_(0) are the initial concentrations of components A and B; and C_A_(t) and C_B_(t) are the concentrations of A and B at time t. S(t) > 0 indicates that selective separation is achieved, whereas S(t) < 0 indicates no selective separation of the two components. 

### 2.3. Ion Selectivity Mechanisms 

Ion transport through an IEM can be divided into five distinctive steps, namely (1) ion transport from the diluted compartment to the membrane surface across a solution boundary layer; (2) partitioning of the ion into the membrane at the surface; (3) ion transport through the bulk of the membrane; (4) egress of the ion on the other side of the membrane surface; and (5) ion transport across a solution boundary layer to a concentrated compartment [16]. The extent of counter-ion selectivity is mostly decided during the partitioning of ionic species into the membrane and the migration of the ions through the membrane matrix and boundary layer in the electrolyte solution. 

**Figure 2:**
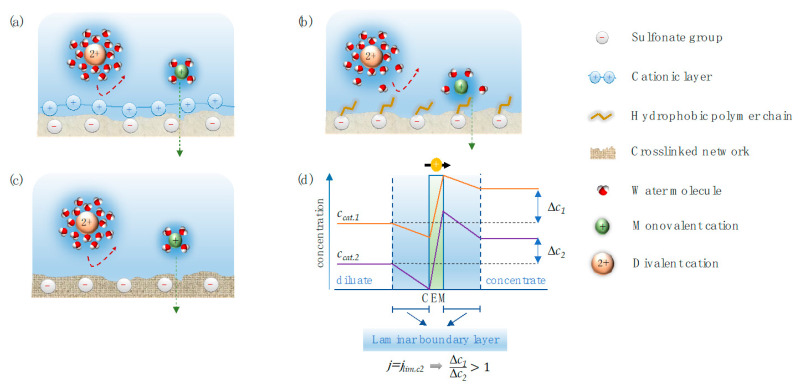
Different parameters of ion selectivity: (**a**) electrostatic barrier effect—imparting a cationic layer on CEM establishes an electrostatic repulsion of the cation with higher valency compared to the monovalent cation; (**b**) dielectric effect—increasing the hydrophobicity of the membrane restricts the passage of cations with higher hydration energy compared to ions with lower hydration energy; (**c**) sieving—introducing a crosslinking agent creates a compact network reduces the ingress of the larger cations, while smaller cations can permeate through the membrane; and (**d**) boundary layer separation—ion depletion within the boundary layer succeeds more or less fast for competing counter-ions based on their concentration, ion characteristics, and the applied current.

In the presence of two or more competing counter-ions, different mechanisms of ion selectivity have been found to be influential on the selectivity of partitioning. One mechanism is identified as an electrostatic barrier effect due to the differences in the electrostatic interaction of counter-ions with the membrane surface (Figure 2a). Generally speaking, electrostatic affinity is more pronounced for counter-ions possessing higher valency or of the larger size of the same valency or lower hydration energy [9,17,18].

Another mechanism is related to the affinity of water of the ion, defined by the Gibbs hydration energy; and the hydrophobicity of the membrane’s surface, affecting the selectivity of ions entering a membrane (Figure 2b) [19]. In this case, selectivity is controlled by the action of the ion partially shedding its water molecules of ions, with ions of lower hydration energy shedding water molecules more easily, allowing the ion to transport through the hydrophobic membrane. 

The steric hindrance may also be a mechanism that governs the selectivity of counter-ions by reducing the partition of larger ions in the case of the dense structure on the IEM, differentiating the ingress rate of the ions, where the dimensions of the hydrophilic entrance into the IEM are typically sub-nanometer, with smaller ions entering much faster than larger ions (Figure 2c) [20]. Partitioning-related mechanisms suggest that the easiness of partition of the counter-ions into the membrane mainly depends on an ions’ size, valency, and hydration energy, as well as the membranes’ properties (i.e., fixed-charge concentration and water uptake) [18,21].

The selectivity of a particular ion is also highly influenced by its mobility in the membrane [18]. The rate of diffusion of the different ions through the bulk of the membrane is largely determined by the size of the ions and their interaction with the fixed ionic groups, as in the process of partitioning ions from solution into the membrane [18]. In addition, the nature of the ionic pathway in the membrane (i.e., hydrophobicity/hydrophilicity, tortuosity, and dimensions) plays a strong role in ion transport and hence selectivity. These parameters are determined by the nature of the polymer that forms the membrane and, more specifically, the morphology of the membrane and its ability to swell in water or resistance to swelling in water. 

Selective separation of counter-ions is also affected by the electrodialysis operating parameters (i.e., current density and flow rate) [4,22,23]. As a consequence of faradaic current flow between two electrodes that necessitates ion flow across an IEM placed between the electrodes, a thin layer is established in the solution at the membrane/solution interface where the ions are depleted. The thickness of the boundary layer is variable, depending on the condition, and influences the rate of ion transport to the membrane surface (Figure 2d) [22]. Control of the current density is important to ion selectivity since ions diffuse at different specific rates across the boundary layer. 

### 2.4. Factors Affecting Ion Selectivity

#### 2.4.1. Ionic Characteristics

The selectivity of counter-ions through an IEM is determined by thermodynamic (affinity of the membrane for the ion) and kinetic (mobility through the membranes) considerations, which are influenced by the specific characteristics of ions. Understanding these characteristics is important to controlling counter-ion selectivity. In this section, the basic characteristics of ions related to ion selectivity are discussed. 

The radii of hydrated ions affect the easiness of the partition and mobility of the ions. Water is polarizable. When salts are dissolved, water molecules interact and align around the ionized components in response to the charge density of the ion. This phenomenon leads to the formation of a hydration shell around the ions [20,24]. Hence, ion transport in solution is correlated with the radii of hydrated ions rather than simply the radii of the bare ion [25,26]. 

**Figure 3:**
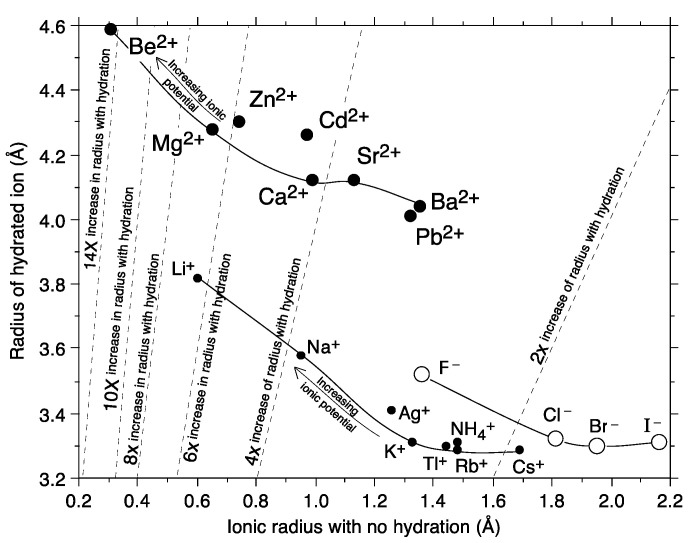
The inverse relationship between radius with hydration and non-hydration. Ions with smaller sizes tend to become more hydrated due to having a higher charge density. The figure is reprinted with permission from “Railsback (15 May 2020) as accessed at http://railsback.org/FundamentalsIndex.html (accessed on 25 May 2023) ”, where Railsback (2020) is “Railsback, L.B., 2020, Some Fundamentals of Mineralogy and Geochemistry. http://railsback.org/FundamentalsIndex.html (accessed on 25 May 2023) ” [29].

The charge density of the ion corresponds to the charge distribution over the volume of the ion (C⋅mm^−3^), as calculated according to the following formula [27,28]:

(3) $$\mathrm{Charge}\;\mathrm{Density}=\frac{\mathrm{ne}}{\left(\frac{4}{3}\right){\pi r}^{3}}$$

where the ionic radii, r, are the Shannon–Prewitt values; e is the electron charge; and n represents the ionic charge (valence number, e.g., $n_{{\mathrm{Na}}^{+}}$ = 1, $n_{{\mathrm{Mg}}^{2+}}$ = 2, etc.). Ions with smaller ionic radii possess a higher charge density, causing an increase in the hydrated radii of the ions. Therefore, the radius of the hydrated ions generally increases as the ionic radius decreases. The inverse relationship between the ionic radius and the hydrated radius of several ions is shown in Figure 3.

Valency is another paramount characteristic of ions, playing an active role in ion partitioning and migration, influencing selective separation. In general, counter-ions of higher valency are more enriched in the membrane network as a result of the stronger coulombic attraction of species with the oppositely charged fixed functional groups in the IEM, prioritizing their permeation compared to counter-ions of lower valency. In contrast, the preferential transport of the ions with higher valency is weakened through the IEMs coated with a surface layer having the same sign as the counter-ions.

The free energy hydration of an ion is an important parameter affecting ion selectivity. The hydration energy is a measure of the strength associated with water molecules, and its inverse (energy of dehydration, endothermic) reflects the easiness of an ion losing waters of hydration. For the latter, ions are required to shed waters of hydration as the ion enters a membrane as part of the ion permeation process [30,31,32]. The energy required to shed water molecules comes from the attraction between the ions and bound oppositely charged groups in the membrane [20,33,34]. The extent to which dehydration occurs depends on the hydration energy of the ions: the lower the free energy of hydration, the easier for the ion to shed water molecules from their hydration shells [31,35]. For the ions that do not shed the water, the hydrophobicity of the membrane determines the permeation process.

Studies investigating the selective separation of Li^+^, Na^+^, and K^+^ (alkali metals); Mg^2+^ and Ca^2+^ (alkaline earth metals); Cu^2+^, Ni^2+^ Co^2+^, Fe^2+/3+^, and Cr^3+^ (transition metals); and Zn^2+^ and Al^3+^ (post-transition metals) are discussed in this work. The ion characteristics are tabulated in Table 1. Briefly, alkali metal ions always possess one valence electron and are hydrated when dissolved in water [36]. They also have very low charge densities, and so do their hydrated size and energy of hydration compared to those of other groups of metals [27,37]. The generalized transport order through the standard CEMs with the fixed sulfonic acid group is reported as follows: transport order alkaline earth metals (Ca^2+^ > Mg^2+^) > transition metals (Cu^2+^ > Zn^2+^) > alkali metals (K^+^ > Na^+^ > Li^+^) > Fe^3+^ [12]. The transport rate of alkali metals lies behind compared to the multivalent metal ions due to their lower affinity for the fixed groups. When the valency is equal, metal selectivity is mainly governed by their hydrated sizes. The exception for Fe^3+^ has been attributed to their slow mobility in the membrane matrix. Despite differences in their transport order, it is unlikely to be sufficient for the selective separation of the metal species through the standard CEMs due to their similar properties. More established counter-ion selectivity can be achieved by specializing in the membrane properties and/or optimizing process conditions, as is discussed later.

#### 2.4.2. Membrane Structural Properties 

**Figure 4:**
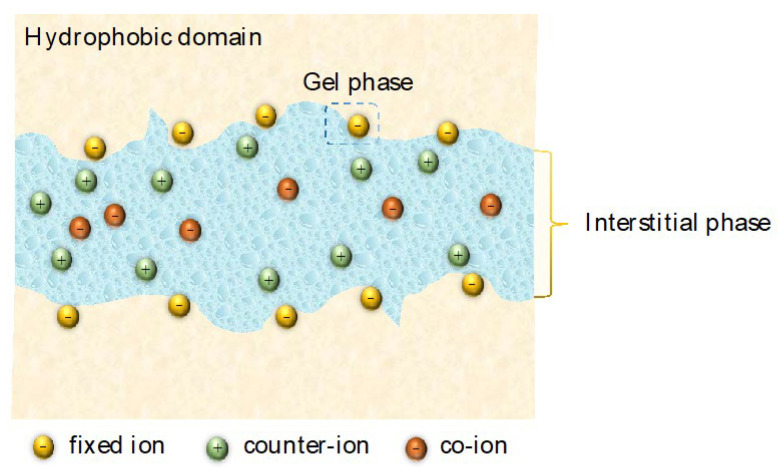
Schematic representation of the CEM structure in accordance with the micro-heterogeneous model. Gel phase, where fixed ionic groups bind to the polymer matrix, and the transport of counter-ions takes place. Interstitial phase, where the transport of co-ions takes place. Hydrophobic domain, devoid of fixed ionic groups. The figure is adapted with permission from [40] (Copyright © 2003, John Wiley and Sons).

The membrane is the core component of electrodialysis. Understanding the structure–property relationships of a CEM is of great importance to achieve enhanced metal ion selectivity. The internal morphology of a CEM can be considered to comprise three phases: (i) an interstitial phase within the bulk of the membrane that comprises pores filled with the electroneutral inter-gel solution; (ii) hydrophobic domains constituted by aggregated polymer chains, devoid of fixed ionic groups; and (iii) a gel phase that serves as an active exchange zone for ions in the solution of pores composed of highly hydrated domains, where the fixed charges bound to the polymer matrix are mixed with the double layer of the solution in the pores [39] (Figure 4). 

In the active region, fixed ions are localized on the pore walls, and this leads to the localization of counter-ions near the pore walls as a result of the Coulomb interaction. The critical distance is termed the Bjerrum length [41], at which the electrostatic attraction between a counter-ion and fixed-charge group is balanced by the thermal energy of the counter-ion. When the distance between counter-ions and fixed groups is smaller than the Bjerrum length, counter-ions condense with the fixed-charge group. If the distance between them is larger than the Bjerrum length, the counter-ions behave as free hydrated ions. The movement of counter-ions through the active region is explained by a hopping mechanism that involves a transfer of ions from one fixed-charge group to another [42]. Hydration shells of the counter-ions are dynamically destructed and reformed during the ion hopping mechanism. 

The interstitial, electroneutral aqueous zone is mainly hosting for the transport of co-ions (i.e., mobile ions having the same charge as the fixed-charge group) because they are repelled by the ion exchange sites in the gel phase. Transport in the interstitial zone is associated with friction ascribed to the water located in the hydration shell of the fixed ions and their counter-ions, which are not free to move within the membrane and will exert drag on ions moving through the pore space [43]. Low degrees of swelling or low ion hydration lead to narrow ionic aqueous domains in the membrane [44]. Hence, constricting the interstitial regions of the membrane generates greater friction and reduces the ingress rate of larger hydrated metal ions more severely than monovalent ions.

All of the above suggest that the species and amount of fixed-charge groups with their distribution in the membrane, as well as the water uptake and the density of the polymer network, have impacts on the partitioning and mobility of the ions, requiring a clear discussion to identify their role on the fractionation of metal ions.

Ion exchange capacity is a measure of the number of fixed charges per unit mass of dry polymer, typically expressed in millimoles per gram. CEMs possess negatively charged sulfonate [45], phosphonate [46], and carboxylate anions [47]. The number of exchange groups in the CEMs can be measured using titration. First, the CEM is equilibrated with HCl and then immersed in NaCl solution to displace H^+^ from the membrane, which is titrated with NaOH solution [18]. The ion exchange capacity can be calculated using the following Equation (4):

(4) $$\mathrm{Ion}\;\mathrm{exchange}\;\mathrm{capacity}\;\left(\frac{\mathrm{mmol}}{g}\right)=\frac{\mathrm{VC}}{W_{\mathrm{dry}}}$$

where V and C are the volume (L) and the concentration (M) of NaOH titration solution, respectively, and W_dry_ is the dry weight of membrane samples (g). Functional groups may be classified as strong or weak electrolytes depending on the degree of the dissociation of their conjugate acid or base [48]. For instance, carboxylic acids are considered weak acids, whereas sulfonic acids are strong acids [49]. The strength of the electrostatic interaction between functional groups and counter-ions is playing a key role in ion selectivity. The sign of the surface charge also plays an acting role in differentiating the partitioning and migration of metal cations of different valences.

The management of water uptake in polymeric membranes is critical for controlling the selectivity performance of the membranes, and this mainly depends on the amount and type of the fixed-charge groups, the nature of the polymeric material, and the characteristics of the ions (i.e., the energy of hydration, hydrated radius, and valency) in the electrolyte solution [18,50]. A high-charge-density membrane takes up a large amount of water and swells, hydrating the fixed-charge groups and their counter-ions [21]. Excessive swelling is generally considered to have an adverse effect on ion selectivity [51]. Conversely, the hydrophobic polymeric material or the degree of crosslinking restricts the amount of water that can penetrate the membrane matrix. All the underlying causes impact the volume fraction of water inside the CEM, influencing the fixed-charge density and tortuosity that affect the partitioning and mobility of the competing metal cations and, hence, the selective separation. Water uptake in the membrane phase can be determined by means of the following Equation (5) [18]:

(5) $$\mathrm{Water}\;\mathrm{uptake}\;\left(\%\right)=\frac{W_{\mathrm{wet}}-W_{\mathrm{dry}}}{W_{\mathrm{dry}}}\cdot 100$$

where W_dry_ and W_wet_ represent the mass of dry and wet membrane samples, respectively.

The density of the polymer network is also an effective parameter for ion separation based on the pore-size sieving considerations [35]. A CEM with high metal ion selectivity should possess uniform and continuous aqueous channels that fit well with the size of target ions. To realize this, membrane architectures are typically designed to possess aqueous molecular features of <2 nm [52]. This feature can be reduced by introducing crosslinking or by coating a crosslinked thin layer with a surface modification [53,54]. The common practice of crosslinking a polymer within a membrane enhances ion selectivity by hindering the passage of bulky ions while allowing smaller ions, but it also may increase the resistance to ion transport. A CEM should possess low electrical resistance, and thus there will be less potential drop during electro-membrane processes [55]. Therefore, applying to crosslink should be optimized depending on the polymers and applications. To decrease the detrimental influence of crosslinking, conducting layers can be introduced onto the membrane surface [56]. 

**Figure 5:**
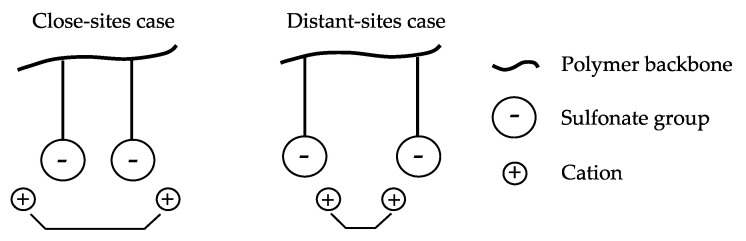
Interaction of anion with adjacent amine groups pendent on the polymer chain. The figure is modified with permission from [57] (Copyright ©1988, Elsevier).

The distance between neighboring fixed groups and their relative spatial configuration can influence the transport rate of counter-ions. According to the distance-of-charge-separation concept, the proximity of neighboring fixed charges is a major contributor to divalent/monovalent selectivity, as illustrated in Figure 5 [57]. When ion exchange sites are widely separated (distant-sites case), monovalent Group I cations are preferred, whereas more closely packed sites (close-sites case) have an increased affinity for divalent Group II cations [58]. A large average distance between two adjacent fixed sulfonate groups eliminates the condensation of the monovalent cations that display higher mobility, whereas divalent ions are condensed, and the mobility ratio of divalent/monovalent ions is observed below 1 [41]. In contrast, the shorter the distance between active groups, the greater the preference for divalent ions over monovalent ions. Nevertheless, fundamental knowledge of the significance of the distance between fixed-charge groups and their distribution in CEMs is still poor, and research is needed to obtain a comprehensive understanding as it applies to electrodialysis.

#### 2.4.3. Ion Transport through the Boundary Layer 

Within tens of micrometers of the membrane surface, the complete mixing of feed and permeate solutions is not obtained, as convection is negligible in this regime [8]. Coupled with the higher counter-ion transport numbers in the membrane compared to the bulk solution, a concentration gradient for each ion is formed. This region of incomplete mixing of ionic species is referred to as a laminar boundary layer, and we discuss its implications on counter-ion selectivity.

The total ionic fluxes, Ji, through the solution and membrane in electrodialysis are described by the Nernst–Planck equation extended by a convective term [59]:

(6) $$\vec{J_{i}}=-D_{i}\left(\nabla C_{i}+z_{i}C_{i}\frac{F}{\mathrm{RT}}\nabla \varphi \right)+C_{i}\vec{V}\;i=+,-,H,\;\mathrm{OH}$$

where $\vec{J_{i}}$, $D_{i}$, $z_{i}$, and $C_{i}\;$are the flux density, diffusion coefficient, charge number, and concentration of ionic species i, respectively; φ is the electrical potential; $\vec{V}$ is the fluid velocity vector; and F, R, and T have their usual meanings. The subscript i may be attributed to the salt cation (+) or anion (–) as well as to H^+^ or OH^-^ ions. The first term on the right-hand side of Equation (4) is proportional to the concentration gradient of ionic species, the second term to the electric field, and the third one to the solvent velocity. The terms represent ionic diffusion, migration, and convection, respectively. Consequently, the transport rate ratio between competing counter-ions will emerge from the ratios of their diffusion coefficients, charge numbers, and concentrations. The key to manipulating counter-ion selectivity through process design is shifting the mass transport control between diffusion and migration. For example, diffusive transport favors monovalent over multivalent ions due to the superior diffusivities of monovalent ions, whereas the migration term is larger for multivalent than monovalent ions, owing to their higher charge numbers. Similar observations can be made for other ion characteristics, such as hydration energy and hydrated sizes. The relative contribution of the three transport mechanisms to ion transport and selectivity depends on process parameters such as flow velocity and current density. 

**Figure 6:**
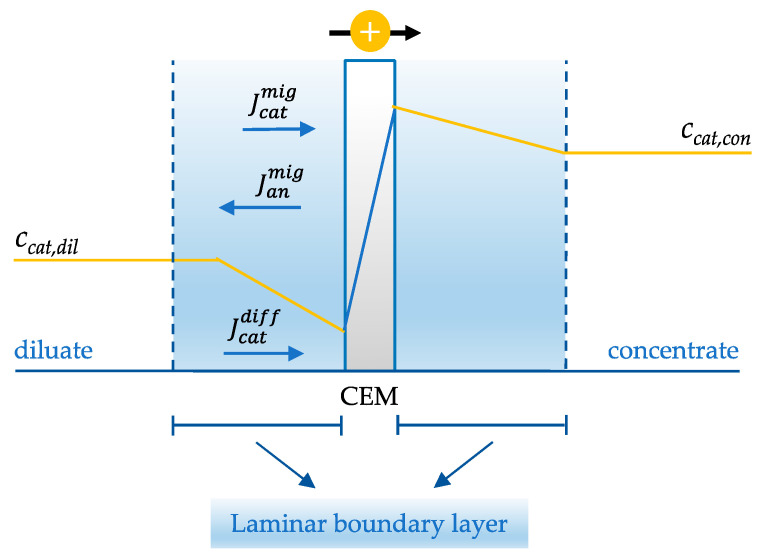
Schematic illustration of the concentration profile of a cation through a CEM and in the laminar boundary layers adjacent to the membrane at the dilute and concentrate side, respectively. The difference in the transport number of ions in the bulk solution compared to the membrane results in a concentration gradient in the boundary layer. The figure is modified with permission from (Copyright ©2010, Elsevier).

In electrodialysis, counter-ions are depleted on the membrane surface at the diluate side and accumulate at the concentrate side. Consequently, ion diffusion in the laminar boundary layer takes place in the same direction of migration for counter-ions and in the opposite direction for co-ions to satisfy the local electroneutrality assumption. The corresponding concentration profile for a cationic species in the laminar boundary layer and through the CEM is illustrated in Figure 6.

**Figure 7:**
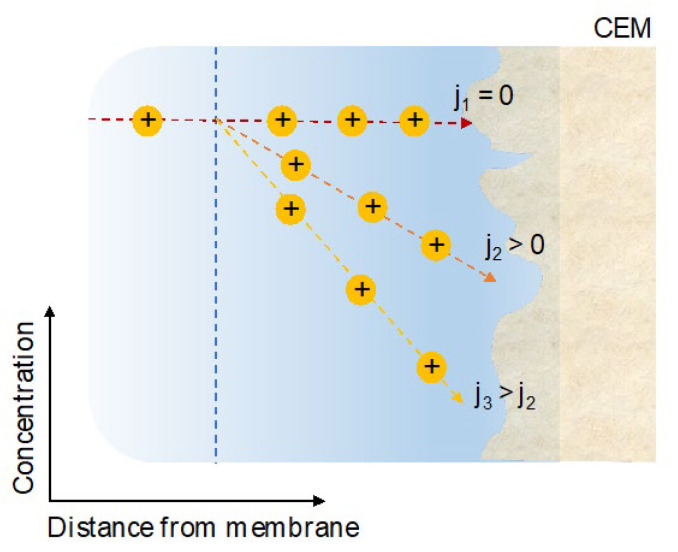
Influence of the current density on the ion concentration profile in the laminar boundary layer at the CEM surface facing the diluate side. The vertical dashed blue lines indicate where the laminar boundary layer ends and the bulk solution begins, in which the solution is completely mixed. The dashed arrows indicate the concentration profiles of cations in solution, which become steeper with the increasing current density, j.

A lowered concentration of ions in the boundary layer increases the ohmic resistance and the energy input requirement to drive the ion transport [60,61]. This phenomenon is frequently termed concentration polarization [62]. With an increasing current density, concentration polarization increases as a result of the depletion of ions at the membrane/solution interface and the formation of steeper concentration profiles, as illustrated in Figure 7. When no current is drawn (j_1_ = 0), no ion transport across the CEM takes place, the boundary layer is negligible, and no concentration gradient of ions is formed. Upon drawing current, ions are depleted, and the rate of ion transport across the boundary layer approaches a steady state if sufficient ions are supplied to the membrane surface by diffusive transport (j_2_ > 0). By increasing the current, the concentration gradient in the boundary layer becomes steeper because the transport rate across the membrane is accelerated (j_3_ > j_2_) [8]. 

When the ion concentration at the membrane surface becomes negligible, the limiting current density is reached, and the ion permeation rate no longer responds to increasing the applied voltage. The limiting current density is dictated by membrane and solution properties, as well as the electrodialysis stack design and operational parameters such as flow velocity of solutions and cell temperature [63], and determines the operational cell resistance and efficiency of the electrodialysis system to a large extent. Electrodialysis systems are, therefore, often operated below the limiting current density to lower power consumption and energy costs [64].

**Figure 8:**
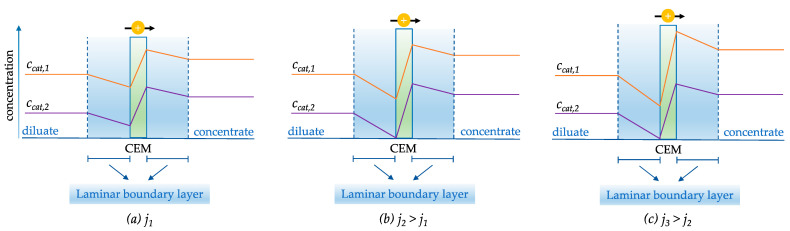
Influence of current density and concentration on selectivity between different counter-ions in solution. The orange and purple curves represent the concentration profiles of two competing counter-ions, Cation 1 and Cation 2, respectively. In (**a**), the applied current density, j_1_, is below the specific limiting current density for both cations. The current density is evenly distributed among the two cations. In (**b**), the current density is increased compared to (**a**), and the limiting current density for Cation 2, j_2_, is reached. In (**c**), the current density is further increased to j_3_. Since the flux density of Cation 2 has reached its maximum value at j_2_, the extra current goes solely into the increased ion flux of Cation 1. Consequently, the selectivity of Cation 1 over Cation 2 increases when operating electrodialysis above the limiting current density for Cation 2.

For multicomponent mixtures, the dependence of the limiting current density on the ion concentration and characteristics allows for partial selectivity between the counter-ions in the electrodialysis process. This is illustrated in Figure 8, where two cations of different concentrations permeate a CEM. In Figure 8a, a current density, j_1_, is applied at which none of the cations is depleted to zero concentration at the membrane surface facing the diluate side. Therefore, the drawn current is distributed evenly across the two ions depending on their charge, according to Equation (1). Figure 8b represents a scenario where the drawn current density corresponds to the specific limiting current density, j_2_, for one of the cations (Cation 2). The current is still distributed evenly, but the concentration of Cation 2 is depleted to zero at the membrane surface facing the diluate side. Therefore, when increasing the current density further to j_3_, as illustrated in Figure 8c, the extra current density will solely result in the increment of the transport rate of Cation 1. In summary, the selectivity for Cation 1 over Cation 2 increases when the current density is bigger than the specific limiting current density for Cation 2. This process-related selectivity mechanism is referred to as the boundary layer separation [5]. It is applicable when the target species of the separation process has a significantly different concentration from competing counter-ions in the solution. In the example, the target ion, Cation 1, has a significantly higher concentration compared to Cation 2. However, boundary layer separation is also useful in the equivalent converse case, where the target ion is present in a very dilute concentration compared to the competing counter-ions. By operating electrodialysis at the limiting current density for the target ion, equivalent to Figure 8b, when Cation 2 is the target ion, the percentage removal (and, hence, the selectivity) is considerably higher for Cation 2 compared to Cation 1 [7].

## 3. CEM Membrane Preparation for Metal Ion Selectivity

The recent developments in membrane fabrication techniques that influence metal cation selectivity are presented, including the following topics, (i) surface modification, (ii) structure and morphology of the polymer, and (iii) incorporating inorganic components into the organic membrane structure.

### 3.1. Surface Modification

Given the importance of ion partition at CEMs, surface characteristics are critical, and modifying membrane surfaces is an effective strategy to influence the membrane’s ability to separate cations. In situ polymerization, the direct coating of charged polymers (i.e., static adsorption, layer-by-layer assembly, and electro-deposition) and surface chemical modification were both explored as a means to modify the surface of the membranes to enhance the selectivity between metal cations [65,66,67].

#### 3.1.1. In Situ Polymerization 

**Figure 9:**
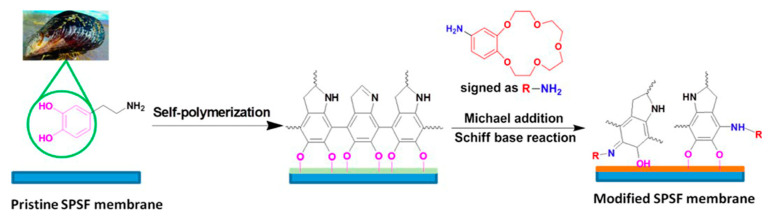
Schematic illustration of the possible co-deposited reaction mechanism of crown ether and dopamine on the surface of sPSF CEM. The figure is reprinted with permission from [73] (Copyright © 2019, American Chemical Society).

In situ polymerization of the monomers is the growth of polymer chains on the membrane surface, which is a means to introduce a synergetic layer. Surface polymerization of dopamine, aniline, and pyrrole to form the corresponding polymer has attracted considerable interest in the last few years [68,69,70,71]. Dopamine, for example, can be oxidized in an alkaline aqueous solution and binds to surfaces via strong adhesive forces [72]. Yang and co-workers [73] fabricated CEMs by modifying sulfonated polysulfone (sPSF) membranes via the co-deposition of dopamine and crown ether, followed by glutaraldehyde crosslinking (Figure 9). Oxygen atoms of the crown ether ring selectively bind specific metal ions through an ion−dipole interaction, which was mimicked by biological host−guest interactions between membrane proteins and ions. The resulting membrane yielded a selectivity of 5.99 in the K^+^/Mg^2+^ and 2.87 in the K^+^/Li^+^ systems, superior to commercial monovalent selective CEM NEOSAPTA CIMS (5.36 and 1.16). Monovalent selectivity was attributed to the pore-size sieving by the compact surface-modified layer and host−guest molecular recognition of the crown ether with K^+^ ions. 

Polyaniline is a cationic conductive polymer that possesses good chemical stability in an acidic medium and can be readily prepared from aniline. Reig et al. [74] reported the synthesis of a monovalent selective CEM by surface polymerization of polyaniline on a polyvinylidene fluoride/sulfonated polyvinylidene fluoride (PVDF/sPVDF) membrane in the presence of p-toluene sulfonic acid or L-valine (2-amino-3-methylbutanoic acid). The dense layer of doped polyaniline resulted in a decreased rate of diffusion of the larger Mg^2+^ cations compared to Na^+^, resulting in doped membranes possessing a higher selectivity for Na^+^ ions (Mg^2+^/Na^+^ = 0.13 for p-toluene sulfonic-acid-doped and Mg^2+^/Na^+^ = 0.09) and for valine-doped compared to Mg^2+^/Na^+^ = 0.63 for unmodified membranes. The hydrophobicity of valine amino acid provided a selective barrier for Mg^2+^_,_ which possesses higher hydration energy compared to Na^+^ ions and is less likely to shed its waters of hydration required to enter a more hydrophobic surface. In similar studies, a thin layer of polyaniline layer grafted on the surface and into the pores of polyacrylonitrile-based ultrafiltration membranes increased the selectivity of Na^+^ ions over Mg^2+^ ions, with monovalent selectivity increasing from 2.15 to 3.98 after surface modification [75]. Polyaniline provides a dense layer with fixed positive charges, resulting in a sieving effect and electrostatic repulsion.

Similar to polyaniline, polypyrrole is a rigid polymer with weakly basic anion exchange groups [71,76]. A crosslinked polyethylene/polystyrene–divinylbenzene-based membrane modified with polypyrrole in the presence of a high oxidant yielded selectivities of Na^+^/Ca^2+^, Na^+^/Mg^2+^, and Na^+^/Cu^2+^ at 5.3, 6.2 and 7.5, respectively. Vazquez et al. [77] applied the galvanostatic modification on CEMs by electrochemical polymerization of polypyrrole without an oxidizing agent. The modified membranes showed a lower passage of divalent cations than monovalent ones. It was ascribed to the fact that Mg^2+^ is a bulkier ion possessing a higher charge and hydration radius than Na^+^, so the hindrance and electrostatic repulsion were more significant. Very recently, Pan et al. [78] carried out the in situ polymerization of pyrrole through subsequent quaternization. The charged surface and crosslinking structure resulted in an improved Na^2+^/Mg^2+^ selectivity of 2.07. 

#### 3.1.2. Direct Coating of Charged Polymers

The coating of surface active agents onto the membrane by using electrostatic attraction and simple immersion is commonly used to modify the surface of membranes [79,80]. Specifically, the selectivity of monovalent cations is more pronounced when an adsorbed layer possessing a positive charge is established on a CEM that carries pendant sulfonates due to a stronger electrostatic repulsion of multivalent counter-ions. For example, Greben and co-workers [81] reported the transport of Mg^2+^, Ca^2+^, and Na^+^ metal ions through a CEM modified with a single layer of chitosan, followed by crosslinking with epichlorohydrin, producing a positively charged surface. After modification, the ion selectivity ratios of Na^+^/Ca^2+^ and Na^+^/Mg^2+^ were improved from 0.45 and 0.84 to 1.82 and 1.79, respectively. Similarly, Jiang et al. [82] modified CEM CR671 by coating polyethyleneimine (PEI) onto the normal grade CEM CR67 to enhance the selectivity of monovalent metal cations. PEI is a positively charged polyelectrolyte where the degree of protonation depends on pH value, and in an acidic medium, it is highly protonated [83]. Monovalent selectivity was improved from 1.06 to 8.61 after the modification of CEM CR671 due to the repulsive forces between the positively charged PEI coating and the multivalent cations. Very recently, Wang et al. [84] developed mussel-inspired membranes through the crosslinking reaction between PEI and different polyphenols on sulfonated polypropylene-based CEM to investigate the selective separation in Na^+^/Mg^2+^ and Li^+^/Mg^2+^ systems. Membranes with relatively high positively charged surfaces resulted in high monovalent selectivity and represented an enhanced stable structure.

Alternating deposition of layers of oppositely charged polymers can be applied as a technique known as layer-by-layer (LbL) deposition to take the adsorption a step further [85,86]. Since the standard CEMs are not selective between counter-ions but special-grade CEMs (e.g., monovalent selective Neosepta CMS and Selemion CSO) are, to some extent, selective to monovalent cations over divalent cations, several research groups employed LbL deposition of polyelectrolytes on commercial CEMs to improve metal ion selectivity [87]. The surface of cationic membranes is mostly terminated by a positively charged polyelectrolyte layer to increase the repulsion toward multivalent species based on Coulomb’s law. 

**Figure 10:**
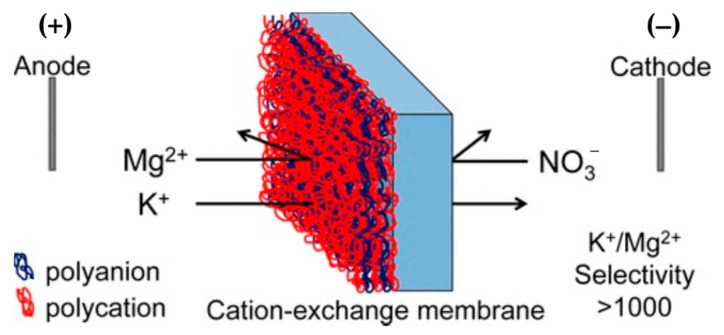
PSS/PAH modified CEM. The figure is reprinted with permission from [92] (Copyright © 2010, American Chemical Society).

Deng et al. [88] modified commercial CEM by the LbL deposition of PEI and poly(acrylic acid) (PAA) crosslinked with epichlorohydrin and reported long-term stable separation efficiency of >0.65 for Na^+^, Ca^2+^, and Mg^2+^ ions. Membranes deposited by protonated PEI resulted in a more hydrophobic surface compared to membranes terminated by PAA, although PAA also played a major role in ion selectivity [89]. Bruening’s team has also shown great interest in the selectivity of monovalent metal cations over divalent ones through LbL deposition of poly(sodium 4-styrene sulfonate)/poly(allylamine hydrochloride (PSS/PAH) polyelectrolyte on different commercial membranes, using electrodialysis, as summarized in Table 2 [90,91,92,93,94,95]. Promising monovalent selectivities were achieved by creating a high electrostatic barrier and dense structure against divalent ions (Figure 10). Selectivity between cations with the same valency has also been investigated via LbL deposition [96]; for instance, (PAH/PSS)_5_PAH multilayers were adsorbed onto the surface of Nafion membranes to separate K^+^ from Li^+^ ions. In electrodialysis, LbL membranes modified at a pH of 2.3 increased K^+^/Li^+^ selectivity from 1.7 to 7. They ascribed improved selectivity to the lowest electrical mobility and partitioning of alkali Li^+^ cations from acidic solutions into the membrane in addition to the obvious effect of Donnan exclusion and sieving. 

LbL shows great promise for enhancing the selectivity of monovalent ions over divalent ions. Notwithstanding, there is a lack of understanding of the structure–property relationships for polyelectrolyte multilayers, as well as their detailed characterization. Therefore, they focused on structure–property relationships for multilayers with respect to hydration, layer thickness, and charge of modified membranes. Rijnaart et al. [87] examined surface layers of PAH/PSS deposition by LbL, using optical techniques and ionic resistance measurements, and found that the selectivity for monovalent ions can be manipulated by changing the ionic resistances of the charged layer. Thicker layers increase the ionic resistance of the membrane. CEMs modified by 13 layers of polyelectrolytes achieved monovalent selectivity as high as 7.8, comparable to the commercial monovalent selective CSO (6.9), owing to an increase in electrostatic repulsion and non-ohmic resistance hindering Mg^2+^ diffusion to the membrane. Furthermore, increasing the number of layers decreased the hydration of the multilayer, thereby achieving higher monovalent selectivity based on size exclusion also. 

**Figure 11:**
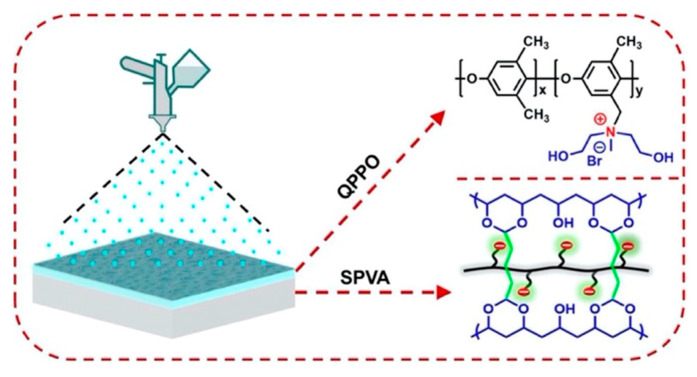
Surface modification of the sulfonated PPO-PVA membrane with the quaternized PPO solution. The figure is reprinted with permission from [101] (Copyright © 2019, Elsevier).

In contrast to the simple immersion coating of substrates in polyelectrolyte solutions, Afsar et al. [97] fabricated modified polyvinyl alcohol-based (PVA) membranes by alternately spray-coating cationic layers of quaternized PPO and anionic sulfonated PPO (Figure 11) [98,99,100] and observed an ion selectivity value of 5 in Li^+^/Mg^2+^ system with high resistance (23.54 Ω⋅cm^2^) due to the formation of a thick, dense layer by the spray-coating method. Subsequently, the number of deposited layers was decreased to one cationic polymer but crosslinked with glutaraldehyde, resulting in a selectivity of 12.7 for Li^+^/Mg^2+^ with a reduced resistance [101].

Significant attention has also been devoted to surface modification by electro-deposition to elevate the ion selectivity without increasing the resistance of the modified membranes. In electro-deposition, a solution (including the modifier) is exposed to an electrical field by applying an electrical potential to the electrodes that attract the oppositely charged modifier, thereby depositing on the membrane surface inside the cell [83]. Greben et al. [102] examined the effect of the electro-deposition of sulfonic cation exchange heterogeneous membranes with chitosan on the selectivity between Na^+^ and Mg^2+^ ions during electrodialysis. The selectivity of the modified membrane with single-layer chitosan was found to be up to 3.3. Hu et al. [103] utilized quaternized, positively charged chitosan by electro-deposition to fractionate H^+^ and metal cations (Zn^2+^ and Al^3+^). The leakage of Zn^2+^ and Al^3+^ decreased from the range 8–9% (unmodified membrane) to around 1% (modified membrane) as a result of more pronounced electrostatic and steric barriers for multivalent cations. Subsequently, Li et al. [104] modified a commercial CEM with chitosan/aniline polymer for the selectivity between H^+^/Zn^2+^, where the Zn^2+^ leakage decreased from 18% to 12% with the increase of the aniline content owing to the formation of side polyaniline chains on the chitosan, resulting in higher electrostatic repulsion to Zn^2+^. However, the leakage remained at a stable level of about 12% despite a further increase in the aniline amount. 

**Figure 12:**
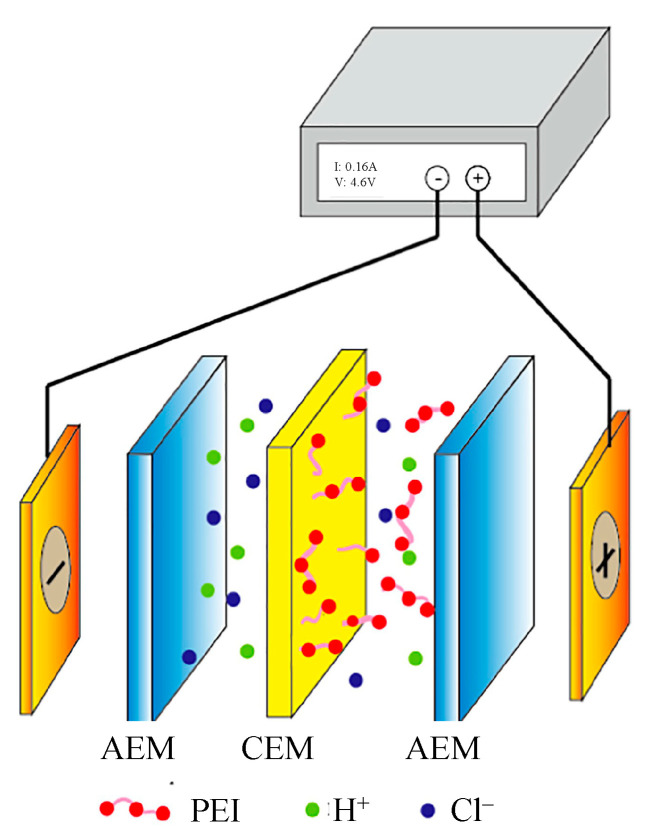
Schematic illustration of membrane modification by electro-deposition. The figure is reprinted with permission from [106] (Copyright © 2019, Elsevier).

Lambert et al. [105] studied the separation of Na^+^ from Cr^3+^, using PEI-modified Nafion^®^ 324. The separation percentages for Na^+^ and Cr^3+^ from the solution were 14% and 42%, respectively. Luo et al. [106] also modified a single layer of PEI on conventional heterogeneous CEMs by electro-deposition but with different molecular weights (Figure 12). The pilot-scale electrodialysis experiments showed that by using a moderate molecular weight of PEI, the selectivity for Ca^2+^/Na^+^ and Mg^2+^/Na^+^ systems was reduced from 0.36 and 0.81 to 0.11 and 0.12, respectively. Very recently, alternate electrodeposition of (PEI/PSS) bilayers on a polyacrylate-based was carried out to fabricate a monovalent selective CEM for Li^+^/Mg^2+^ separation [107], which revealed a selectivity of up to 4.59 and temporal retardation on divalent cations, while a quick passage for monovalent cations occurred through polyelectrolyte bilayers.

#### 3.1.3. Surface Chemical Modification 

**Figure 13:**
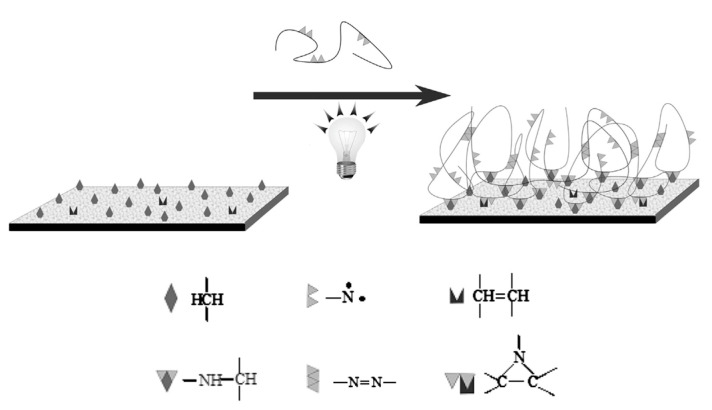
CEM modification by the covalent immobilization and self-crosslinking of the chitosan. Schematic illustration of membrane modification by electro-deposition. The figure is reprinted with permission from [112] (Copyright © 2013, Elsevier).

The chemical modification of membrane surfaces with different functional groups is an effective method to introduce desired surface properties, including photo-induced immobilization, diazonium-induced anchoring, and the formation of sulfonamides [108,109,110,111]. Wang et al. [112] modified the surface of a conventional CEM by photo-induced covalent immobilization and self-crosslinking of azide-functionalized chitosan for Na^+^/Mg^2+^ and H^+^/Zn^2+^ systems (Figure 13). Surface modification reduced the leakage of Zn^2+^ and Mg^2+^ ions by 27.4% and 62.4%, respectively, due again to increased charge intensity and compactness of the surface layer, which amplified electrostatic repulsion and sieving of bulkier cations. Liu et al. [113,114] used the photosensitive 4,4-diazo-stilbene-2,2-disulfonic acid disodium salt to photo-crosslink the membrane surface to improve the selectivity and durability of the membranes [113]. 

**Figure 14:**
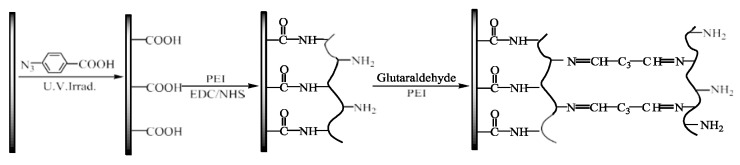
CEM modification by the covalent immobilization of PEI. The figure is reprinted with permission from [116] (Copyright © 2015, Elsevier).

A spontaneous grafting of aryldiazonium salts is a way to form covalent bonds at the membrane surface [115]. A three-step modification comprising (i) diazonium-induced carboxyl group grafting under UV-light irradiation, (ii) grafting of PEI by amidation of the surface carboxyl groups, and (iii) glutaraldehyde-crosslinked PEI multilayers is represented in Figure 14 [116,117]. A series of electrodialysis studies on H^+^/Zn^2+^ and Na^+^/Mg^2+^ cells were conducted to evaluate the monovalent selectivity, which was reported to increase after immobilization by decreasing the divalent ion leakage rate to < 10% of the pristine membranes [116]. Using PEI-modified membranes reduced the Mg^2+^ and Zn^2+^ leakage by 75% and 81%, respectively, of their original value [117,118]. Modified membranes displayed stable performance after 60 days but exhibited increased membrane resistance.

The formation of sulfonamide bonds is a pathway to improve the stability between the membrane surface and the active layer [119,120,121,122], wherein a solution of an amine is reacted with a membrane possessing sulfonyl groups. Sata et al. [123] used sulfonamide bonding between PEI and a commercial CEM membrane to investigate the selectivity between Na^+^ and Ca^2+^ ions. A monovalent selectivity of 3.3 was achieved. Similarly, Li and co-workers also created sulfonamide bonds with surfactant poly-quaternium-7 and the surface of a chlorosulfonated commercial CEM [124]. The resulting membrane possessed cationic surface groups and exhibited improved selectivity toward H^+^ in the electrodialysis by decreasing Zn^2+^, Ca^2+^, and Mg^2+^ leakage from 22 to 14%, 53 to 38%, and 82 to 33%, respectively.

Interfacial polymerization is yet another effective way to create a thin layer through membrane-covalent bonds with a membrane. Hou et al. [125] reported the synthesis of an ultrathin polyamide selective layer on a negatively charged polyacrylonitrile porous substrate via the interfacial polymerization of three water-soluble monomers (ethylenediamine, tetraethylenepentamine and PEI) with organic-soluble monomer, yielding the selectivity between Na^+^ and Mg^2+^ of 3.3 when ethylenediamine was used. Li et al. [126] fabricated a series of PVA-based monovalent anion-selective membranes by depositing a thin electronegative layer with a loose structure on the surface of the membrane through interfacial polymerization of 4,4′-diaminodiphenylamine-2′-sulfonic acid and trimesoyl chloride. Membranes possessing the longest hydrophobic alkyl side chain and largest 4,4′-diaminodiphenylamine-2-sulfonic acid concentration resulted in the highest selectivity value (6.3) but the largest membrane resistance. 

**Figure 15:**
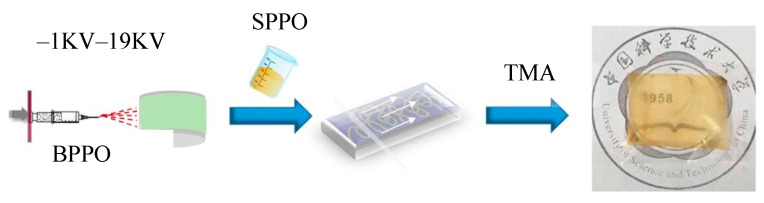
Schematic illustrations for nanofibrous composite membranes. The figure is reprinted with permission from [128] (Copyright © 2017, Elsevier).

Attaching quaternary amine functionalities may also be used to form covalent linkages between the membrane and a surface layer. For instance, Yao et al. [127] immersed a CEM into a chitosan solution. Subsequently, they carried out post-quaternization using the reaction of glycidyl trimethyl ammonium chloride with amine groups of chitosan and performed electrodialysis of H^+^/Zn^2+^. The modified membranes improved the separation ability between H^+^ and Zn^2+^ by 10×, but the membrane possessed a high resistance (60 Ω⋅cm^2^). Hou et al. [128] prepared nanofibrous composite membranes by impregnating bromomethylated poly(2,6-dimethyl-1,4-phenylene oxide) (BPPO) electrospun nanofibrous mats into sulfonated PPO solution (Figure 15), and the resulting membrane was immersed in a trimethylamine solution to introduce positively charged quaternary ammonium groups. A selectivity of 1.6 was achieved in an electrodialysis cell with a feed of NaCl/MgCl_2_. Pang et al. [129] prepared a cation-selective membrane with a commercial CEM by sequential surface polymerization of aniline and quaternization with methyl iodide, and the quaternized polyaniline membranes exhibited a Na^+^/Mg^2+^ selectivity of 4.1 and a Li^+^/Mg^2+^ selectivity of 1.75. The density of the cationic surface charge was tuned by controlling the degree of quaternization. It was observed that, with an increase in quaternization, the flux of both monovalent cations and divalent cations decreased due to the increased repulsive force on both cations.

### 3.2. Structure and Morphology of the Polymer Matrix

Tuning the structure of the CEM matrix is an effective approach to enhance metal ion selectivity; incorporating functional groups into the polymer matrix, either prior to the formation of the membrane or by post-fabrication, can alter its hydrophilic character, porosity, charge density, and swelling characteristics—parameters that remarkably influence the selectivity of counter-ions. 

#### 3.2.1. Physical Blend of Polymers

In principle, blending is a simple process in which at least two polymers are physically mixed before membrane formation. One of the polymers must possess fixed-charge groups or functionality, leading to fixed-charge groups. For CEMs, sulfonated polymers are typically used, and their properties are well-documented to blend with a second polymer to enhance the overall properties of the fabricated membrane [130]. Tas et al. [131] prepared CEMs by blending sulfonated poly(ether ether ketone) (sPEEK) with poly(arylene ether ketone) possessing units of crown ether to study selective K^+^ ion transport over Li^+^ ions. Selectivity was improved by ~4× compared to pure sPEEK membranes, and this was ascribed to the enhanced hydrophobic character of the membrane. Sulfonated arylene main-chain polymers are attractive materials for many electrochemical applications [132]. Poly(arylsulfone)s have been used in the preparation of CEMs, offering strong mechanical, thermal, and chemical stability [133,134]. Gohil et al. [135] studied a blend of sulfonated poly(ether sulfone) (sPES) and sPEEK for cation selectivity of Na^+^ in the presence of Ca^2+^ and Mg^2+^ ions. The selectivity values of divalent cations decreased with a decrease in the weight fraction of sPEEK content (from 100% to 50%). Below 50% content, not all ion exchange groups were available for ion transport, whereas, above that range, the membranes swelled excessively. 

Hydrophobic polymer PVDF may be sulfonated to possess good mechanical strength, chemical resistance, and thermal stability but still has a low affinity with water even after sulfonation because of its highly hydrophobic nature [136]. Farrokhzad et al. [137] prepared hybrid CEMs composed of a blend of PVDF, sPVDF, and doped polyaniline, with the latter serving as an additive. The selectivities of Ca^2+^/Na^+^ and Mg^2+^/Na^+^ were found to be 2.9 and 2.2, respectively. It is postulated that a strong affinity between the negative exchange sites of the membrane and divalent cations reduced the number of sites used by the Na^+^ cations. Therefore, increasing the sulfonic groups in the membrane structure enabled more sites to be used by divalent cations than monovalent ones, which increased the divalent selectivity.

#### 3.2.2. Micro-Phase Separated Structure from Chemically Grafted Polymer

As ion selectivity is a strong function of the affinity of ions for fixed-charge groups in the membranes [138] and Gibbs hydration energy of an ion affects the affinity with the membrane, especially through hydrophobic ones, the hydrophobic domains of the membrane can hinder the permeation of strongly hydrated ions. In contrast, less hydrated ions can easily permeate through the membrane [9,17,19]. Side-chain-type CEMs are believed to offer the prominent hydrophilic/hydrophobic discrimination between the flexible functionalized segments and unfunctionalized backbones, yielding a micro-phase separated structure that favors the high mobility of counter-ions on conductive groups [139,140]. 

**Figure 16:**
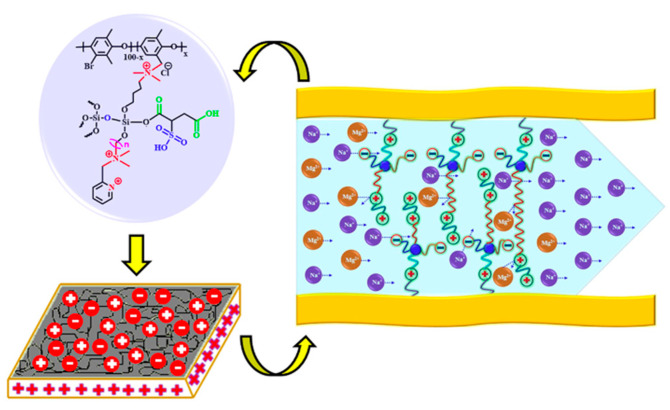
The effect of hydrophobicity and zwitterion structure on selectivity. The figure is reprinted with permission from [143] (Copyright © 2020, Elsevier).

CEMs with different hydrophobic alkyl spacers and zwitterion structures have been reported for monovalent/divalent metal cation separation. He et al. [141] synthesized functionalized monovalent cation selective membranes containing zwitterionic side chains with two quaternary ammonium groups and one sulfonic acid group. In the electrodialysis process, the resulting membrane showed Na^+^/Mg^2+^ and H^+^/Zn^2+^ selectivity of 7.4 and 23.5, respectively. Irfan et al. [142] also utilized monovalent selective CEMs with a zwitterion structure comprising three quaternary ammonium groups, two carboxylic acids, and one sulfonic acid group. Synthesized quaternized poly(6-dimethylamino-1-hexanol-N-2,3-dimethyl phenyl oxide) from BPPO and 6-dimethylamino-1-hexanol was used in the membrane preparation. The modified zwitterion membranes containing hydrophobic and hydrophilic domains were subjected to heating to crosslink the unreacted groups within the structure. The selectivities of the fabricated membranes were 58.4 and 16.5 for Na^+^/Mg^2+^ and Li^+^/Mg^2+^ systems, respectively. In theory, the sulfonic and carboxylic acid groups act as continuous carriers for cation transmission, whereas the quaternary ammonium group improved the separation of monovalent and divalent cations through electrostatic repulsion. Consequently, the transfer of monovalent ions inside the membrane was improved because of electrostatic attraction with negatively charged fixed groups, whereas Mg^2+^ ion permeability was decreased owing to an increase in the electrostatic repulsive effect in the membrane matrix. Moreover, the content of the crosslinking agent and heat treatment contributed to the ion selectivity by the size-based exclusion of highly hydrated ions. More recently, selective CEMs from quaternized PPOs containing different lengths of alkyl spacers were developed and grafted directly to the nitrogen-centered cations connected to hydrophilic carboxylic and sulfonic acids groups (Figure 16) [143], and the resulting membranes with longer hydrophobic alkyl spacer exhibited a high selectivity of 25.26 in the Na^+^/Mg^+2^ system, suggesting that membrane hydrophobicity effectively improved the transport of monovalent cation through the membrane. 

A different application of membrane architecture with nanophase separation was also reported. Wang et al. [144] produced comb-shaped sPEEK membranes with long alkyl (butyl and octyl) side chains for electrodialysis. The comb-shaped membranes prepared with 30% substitution of octyl side chains showed a high H^+^/Fe^2+^ selectivity of 32.13. It was presumed that well-defined hydrophilic/hydrophobic separation of the comb-shaped membranes by increasing the degree of substitution and the length of the alkyl side chains enhanced the transport of H^+^. Moreover, the lower ion exchange capacity and water uptake resulted in a lower swelling ratio. Therefore, lower Fe^2+^ leakage was observed owing to the larger hydrated radius of Fe^2+^ than that of H^+^ during the electrodialysis process. Lin et al. [145] recently constructed novel cationic conductive biomimetic nanochannels by ionically crosslinking sPEEK and quaternized PPO. Two electrolyte ionomers with opposite charges were used for ionically crosslink membranes with other elementary pore units, forming an interconnected structure. The resulting architecture caused the size-sieving and electrostatic repulsion effects, yielding a high cationic selectivity of 7.91 for K^+^/Mg^2+^.

### 3.3. Inorganic–Organic Hybrid CEMs

There are several routes to synthesize hybrid CEMs, including intercalation, blending, in situ polymerization, and molecular self-assembly [145]. Utilizing inorganic particles or fillers has been one of the popular ways to enhance both ionic flux and the selectivity of the membranes [146]. Hybrid CEMs consisting of a polymeric matrix and inorganic fillers have received much attention in recent years due to their combined advantages from both organic and inorganic materials. While polymers provide bulk phases with adjustable charge density and processing ability, inorganic particles dispersed into the membrane bulk can enhance thermal and mechanical stability, as well as desired electrochemical properties. 

Inorganic–organic hybrid CEMs were first developed in the late 1990s, using a sol–gel process for applications in severe conditions, such as high temperatures and strongly oxidizing circumstances [147,148]. Kumar et al. [149] used the sol–gel method to prepare PVA-silica-based selective CEMs through grafting functional groups (–SO_3_H) on the inorganic segment (silica) under acidic conditions. For the Si-65%-modified membrane, the electro-separation of Na^+^ from Ca^2+^, Mg^2+^, and Fe^3+^ was 3, 5.8, and 9.5, respectively. It was explained by the fact that, after the inclusion of silica in the PVA matrix, the accessibility of bulkier counter-ions to the fixed ionogenic sites on the membrane matrix became more difficult for a more compact/rigid membrane matrix. Furthermore, the formation of hydrophilic channels by incorporating silica in the PVA matrix and functionalizing the inorganic part resulted in a higher interaction with Na^+^ among all cations. 

Srivastava et al. [150] prepared CEMs by homogeneously embedding BaCO_3_ nanoparticles into a sulfonated PES matrix. The selectivity between Na^+^ and Mg^2+^ was obtained as high as 8.8, which was attributed to the Mg^2+^ retentive nature of the embedded BaCO_3_ nanoparticles and the rigidity of modified membranes, allowing the electro-migration of smaller ions (Na^+^) across the membrane, and retained relatively larger Mg^2+^ cations. More recently, Golubenka et al. [151] fabricated hybrid CEMs by incorporating amorphous zirconium phosphate into the bulk and the surface layer of heterogeneous CEM RALEX^®^ CM commercial membrane. It was shown that the surface-modified membranes improved the selectivity to 0.7 (Ca^2+^/Na^+^) by decreasing the mobility of Ca^2+^ up to 4.6-fold. However, relatively high ionic resistance of the resulting membranes was obtained depending on the thickness of the modified layer, which might increase the energy loss and decrease the current efficiency. 

Metal oxides or metal particles can also be incorporated into membrane matrices as different types of inorganic components. An et al. [152] used 2-acrylamido-2-methylpropane sulfonic acid and synthesized a series of novel inorganic–organic crosslinked membranes by UV irradiation with various SiO_2_ loadings. The selectivity of all prepared membranes increased gradually at low silica contents. When the silica content was 4 wt.%, the selectivities of 1.70, 5.84, and 25.67 were obtained in K^+^/Na^+^, K^+^/Mg^2+^, and K^+^/Ca^2+^ systems, respectively. They deduced that, at low SiO_2_ contents, the silica might be fully combined with the membrane matrix, resulting in less effective binding sites. This, in turn, reduced the binding efficiency of multivalent cations and facilitated the migration of monovalent cations through the membrane, but an area resistance of 140.11 (Ω⋅cm^2^) was reported. Thakur et al. [153] also used poly(2-acrylamido-2-methyl-propane-sulfonic acid) with PVA in the preparation of the crosslinked CEMs, followed by developing polymer–metal composites for the separation of monovalent cations from divalent cations. The prepared membrane surface was modified with copper loading in a single step by the ion exchange process where the metallic counter-ion and reducing agent (hydrazine hydrate) diffused through both sides of the membrane matrix, forming a homogenous and thin metallic layer. The loaded metal particles showed improved surface compactness, which restricted the transport of Ni^2+^ and Zn^2+^ ions without any significant deterioration in the transport of Na^+^ ions.

**Figure 17:**
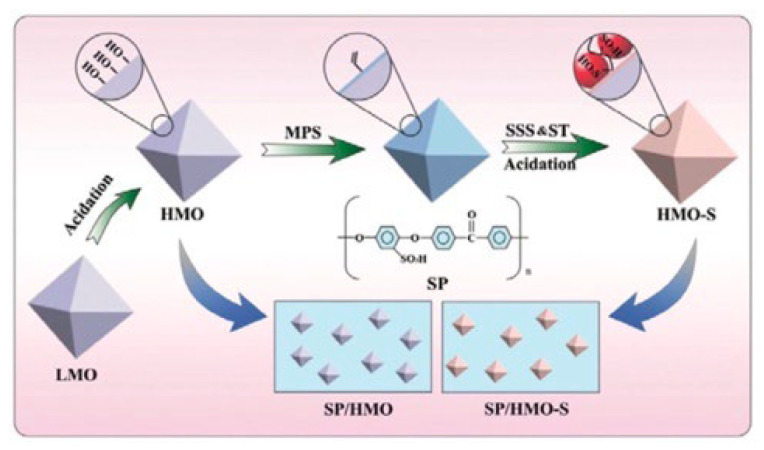
The preparation procedure of lithium-ion sieves and their hybrid membranes. The figure is reprinted with permission from [154] (Copyright © 2018, John Wiley and Sons).

Additionally, the inorganic fillers can be functionalized with desirable groups through chemical or physical modification to construct continuous transfer pathways with additional transporting sites and to improve compatibility with the organic matrix for enhanced mechanical stability [154]. Zhang et al. [154] developed Li^+^-selective CEMs by dispersing two different kinds of lithium-ion sieves into an sPEEK matrix, including acidified lithium-ion sieves and their sulfonation compound, as shown in Figure 17. They demonstrated enhanced Li^+^ selectivity (Li^+^/Mg^2+^ = 3.1 and Li^+^/K^+^ = 1.3) due to the ion-sieve effect of the channels, which weakened the migration of Mg^2+^ and K^+^ but allowed Li^+^ ions to transport efficiently through the channels. The resultant hybrid membrane also reduced the area resistance from 8.0 to less than 6.0 Ω⋅cm^2^. 

MOFs can also be used to construct transfer pathways with tunable pores and functional groups for ion fractionation. They have attracted considerable attention because of their organic components, which can form synergism with polymers [145]. Zhang et al. [155] prepared hybrid membranes with a polyvinyl chloride matrix and six different MOFs (ZIF-8, UiO-66, HSO_3_-UiO-66, HKUST-1, MOF-808, and SO_4_-MOF-808) via the casting method to separate Li^+^ from Mg^2+^ in the solution. The performance results represented that polyvinyl chloride membrane containing sulfonated Zr-MOFs, HSO_3_-UiO-66 showed high stability after a 78-day test in saline solution with the separation ratio of Li^+^/Mg^2+^ > 4. It was reasoned that when the pore size of MOFs became smaller, hydrated Li^+^ ions removed water molecules easier for entering the pore than hydrated Mg^2+^ ions. Moreover, the sulfonate groups anchored in the MOFs delayed the Mg^2+^ transfer due to the strong binding affinity. Recently, Abdollahzadeh et al. [156] introduced a highly tunable design concept to fabricate monovalent cation selective membranes with asymmetric sub-nanometer pores. Relatively high selectivity ratios of 84 and 80 for K^+^/Li^+^ and Na^+^/Li^+^ were represented. However, it should be noted that pore defects are generally difficult to avoid in the growth of MOF films, and it is still challenging to obtain full coverage of MOFs on supporting materials with a large area. Moreover, membranes based on MOFs are difficult to develop because of poor chemical stability and mechanical strength [157]. 

**Figure 18:**
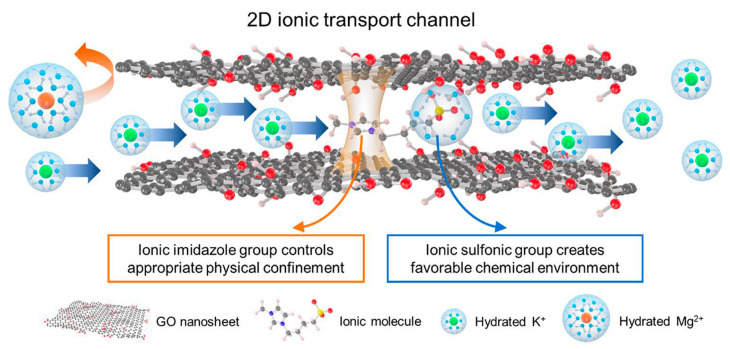
Biomimic 2D ionic transport channels for efficient ion sieving. The figure is reprinted with permission from [160] (Copyright © 2021, American Chemical Society).

Graphene has been used for the selective separation of monovalent ions due to its good mechanical strength and chemical stability [158,159]. Zhang and co-workers were inspired by biological ion channels and designed biomimic two-dimensional ionic transport channels based on a graphene oxide membrane for efficient ion sieving [160]. The ionic imidazole group tuning the appropriate physical confinement of 2D ionic transport channels corresponds to the confined cavity structures of the biological selectivity filter, and the ionic sulfonic group forming a favorable chemical environment of 2D ionic transport channels denotes the affinitive binding sites along the selective filter (Figure 18). The resulting ionic graphene oxide membrane represented K^+^/Ca^2+^, K^+^/Cu^2+^, K^+^/Mg^2+^, and K^+^/Fe^3+^ selectivities of 6.44, ~8.93, ~9.11, and ~28.29, respectively, with a faster K^+^ transport rate compared with the pristine graphene oxide. This performance was explained by the fact that while the sulfonic group accounted for affinity with hydrated K^+^ ions, the ionic imidazole group suppressed the transport of hydrated divalent ions, providing physical confinement of 2D ionic transport channels. Moreover, due to the high binding energy between the ionic sulfonic group and a water molecule, the permeation of monovalent hydrated ions with hydration shells of the ionic-graphene oxide membrane was significantly improved, while multivalent metal cations underwent a steric hindrance exclusion effect. In a recent study by Huang et al. [161], an interlayer charge-regulated graphene-oxide/PEI membrane was developed for the separation of monovalent cations from divalent ones in a homemade ion permeation device. The introduction of the positively charged PEI into the interlayer of negatively charged GO laminates created an enhanced sieving effect, and the Donnan exclusion resulted in a selectivity for K^+^/Mg^2+^, Na^+^/Mg^2+^, and Li^+^/Mg^2+^ of 33.8, 27.0, and 21.9, respectively.

## 4. Impact of Process Parameters on Metal Ion Selectivity

Ion selectivity is also significantly influenced by process conditions such as solution concentrations and composition, flow rate, and current density [162]. Therefore, it is paramount to integrate process design with membrane material considerations to optimize ion selectivity in electrodialysis. The main process control factors for favoring the transport of one counter-ion over another in electrodialysis are the boundary layer thickness and current density. According to the literature, divalent cations usually have a higher affinity to the CEM than monovalent cations due to their higher valency [163,164,165,166]. When the limiting current is not reached for any of the ions, the competitive transport between mono- and multivalent ions is primarily governed by the partitioning with CEMs, as affirmed by various studies in the field [164,167,168,169,170]. However, monovalent ions are smaller than multivalent ions and, therefore, have a higher diffusive transport rate in the laminar boundary layer. Table 3 gives an overview of recent studies on how the stack design and process conditions influence the separation efficiency between counter-ions in electrodialysis.

### 4.1. Boundary Layer Thickness

In general, a thicker boundary layer provides an advantage to monovalent over divalent ions due to the increasing importance of diffusive transport through the boundary layer. Conversely, the selectivity for divalent over monovalent ions increases for common IEMs when the bulk electrolyte is thoroughly mixed, and the selectivity is primarily determined by the ion affinity to the membrane. When using monovalent selective IEMs, the situation becomes more complex. Divalent ions get repelled from the membrane surface, building up an accumulation and hindering the passage of other ions. Additionally, the effect of Donnan exclusion is weakened [173,183]. Therefore, mixing plays a decisive role in electrodialysis with counter-ion selectivity. 

The boundary layer thickness decreases by increasing the flow rate at which the feed solution travels through the electrodialysis stack. In general, the limiting current density increases with an increasing flow rate because the diffusion layer is thinner, and the transport rate of ions to the membrane surface is accelerated. In the cases where the target cation is present in trace concentrations, increasing mixing will increase the target cation’s limiting current density and lower the time and/or space requirement of electrodialysis. Kim et al. [22] studied the effect of the flow rate on selectivity between Ca^2+^ and K^+^ in electrodialysis with standard commercial CEMs. They found that increasing the flow rate accelerated the relative separation rate of Ca^2+^ over K^+^. While the CEM is more selective toward Ca^2+^ due to its greater ionic charge, the boundary layer is favorable for K^+^ transport due to its greater diffusivity, approximately three times that of Ca^2+^.

Nie et al. [174] investigated the influence of the flow rate on the Li^+^/Mg^2+^ separation performance by using monovalent selective CEMs. Increasing the flow rate increased the mass transport of Li^+^ but had no significant effect on Mg^2+^. Consequently, the selectivity for Li^+^ over Mg^2+^ was enhanced by increasing the flow rate. Mg^2+^ was considerably more abundant than Li^+^, with mass ratios from 21 to 422 between the cations. The increased flow rate might have induced back-mixing of Mg^2+^ that accumulated at the surface of the monovalent selective CEMs and thereby hindered the passage of Li^+^. At the same time, the low Li^+^ concentration could have led to the depletion of Li^+^ ions at the membrane surface, limiting Li^+^ transport diffusion. By increasing the flow rate, i.e., decreasing the boundary layer thickness, more Li^+^ ions are available at the membrane surface, increasing the transport ratio of Li^+^ versus Mg^2+^. Hence, concentration effects on the mass transport through the boundary layer need to be considered when evaluating the implications of varying the flow rate.

Apart from increasing the flow rate, which might be limited by the pressure drop in the electrodialysis cell, another common approach to enhance mixing is to install convection promoters near the membrane surface, such as spacers. The geometry of such spacers, such as the length-to-height ratio, can be optimized to maximize the mass transfer [185]. However, non-conductive spacers significantly increase the resistance in electrodialysis, as they block off parts of the membrane area. Sano et al. [186] tested porous spacers made of ceramic foam for electrodialysis as an alternative to meshed spacers. The ceramic foam’s hydrodynamic mixing behavior assisted in suppressing concentration polarization, consequently allowing for a higher limiting current density. The experimental results indicate a notable increase in the limiting current density when employing porous spacers in contrast to meshed spacers, as observed in a comparative analysis of setups lacking spacers. A higher limiting current density allows for higher currents to be applied without compromising the current efficiency. Consequently, a higher mass transport rate of the target ion can be achieved.

### 4.2. Current Density

Current density controls the mass transport regime and, hence, impacts ion selectivity. At under-limiting current densities, the ion selectivity is determined by the transport rate of the competing ions in the membrane, while at over-limiting current densities, the ion selectivity is predominantly determined by the transport properties of the ions in the boundary layer [173,187,188]. For non-selective IEMs, it is reported that a lower current density leads to a more complete reduction in the concentration of divalent over the monovalent cations [22,179,181], and this is ascribed to the positive correlation between valency and membrane affinity. However, with the increasing current density [189], the kinetic control is shifted from the membrane phase to the boundary layer, leading to a loss of membrane selectivity, but it usually benefits monovalent ions due to their generally higher diffusivities [189]. Concentration polarization is the effect that impedes ion transport through the membrane and transfers kinetic control to the boundary layer. However, the change in ion selectivity manifests itself well before the current–voltage curves shift from an ohmic to non-ohmic behavior [190]. Similar findings have been reported for selectivity between like-charged cations: Ozkul et al. [184] modeled the mass transport of Na^+^ and K^+^ through a CEM, considering contributions from electromigration, diffusion, and convection. Electromigration and convection were dominant at the beginning of the experiments, promoting selectivity for K^+^, which has a higher affinity to the membrane. With increasing concentration polarization, diffusion became more significant, increasing the mass transport of Na^+^ over K^+^.

Roghmans et al. [183] compared the dependence of ion selectivity between Ca^2+^ and Na^+^ on current density for a monovalent selective and a normal-grade CEM. While the common CEM met the expectation of increasing Na^+^ selectivity with increasing current, the reverse relation was observed for the monovalent selective CEM. Ambiguous findings are reported for the influence of current density on the selectivity between monovalent and divalent ions, using monovalent selective membranes. Zhang et al. [172] compared the selective removal of Na^+^ over Ca^2+^ (concentration ratio Ca^2+^/Na^+^ = 44) and Na^+^ over Mg^2+^ (concentration ratio Mg^2+^/Na^+^ = 103) by testing two different monovalent selective CEMs at two current density levels. Increasing the current density from 40 to 100 A/m^2^ did not significantly alter the final Na^+^ concentration. However, the permeance of both Mg^2+^ and Ca^2+^ increased significantly. Other studies confirm this current density dependence for Na^+^ over Mg^2+^, also when using a 1:1 concentration ratio of monovalent to divalent ions [173,178]. Xu et al. [63] reported that the transport of divalent cations through monovalent selective CEMs was increased with the increasing the current density. Conversely, Nie et al. [174] found that a higher current density increased the selectivity between Li^+^ over Mg^2+^ ions, using a monovalent selective CEM and mass ratios for Mg^2+^/Li^+^ between 21 and 422.

The inconsistent findings about the influence of current density on the selectivity between monovalent and divalent cations using monovalent selective CEMs can be explained by a model developed by Gorobchenko et al. [191] that identifies three current control regimes: (1) at low current densities, ion diffusion to the membrane surface is fast enough to avert ion depletion, leaving the transport rate control to the substrate layer, which favors multivalent over monovalent cations; (2) with increasing current density, the control is shifted to the modified layer, promoting selectivity for monovalent over divalent cations; and (3) close to the limiting current density of the mixture, the membrane selectivity is negligible, and the counter-ion selectivity is determined by the diffusion in the boundary layer, leading to a loss in ion selectivity. For the selective transport of Ca^2+^ and Na^+^ through a CEM modified with a surface layer for monovalent selectivity, the model predicted an increase of monovalent selectivity with the current density at lower currents until passing through a maximum followed by a drop in selectivity [191], and this agrees with former studies on ion exchange membranes [192]. In consequence, the dependence of the ion selectivity on the electric current density has a maximum, and the findings of different groups can differ according to the current regime they applied. The established model suggests that the maximum selectivity for Na^+^ over Ca^2+^ was reached close to the limiting value of the Na^+^ ion flux. Zimmermann et al. [7] exploited this concept for separating monovalent from divalent ions with monovalent selective IEMs, where operating electrodialysis according to the limiting current density of the target ion yielded greater selectivity compared to working at higher or lower currents.

Recent research suggested that applying a pulsed electric field during electrodialysis can help mitigate the loss of ion selectivity at increased current densities [23,193]. Instead of drawing a constant current, a non-stationary electric current regime is imposed, where pulses of current are followed by pause lapses. The pulsed electric field effectively increases ionic mass transfer and decreases concentration polarization, mitigating membrane fouling and scaling. During the pause lapse, the boundary layer degenerates, and the ionic concentration gradients flatten, shifting the kinetic control back to the membrane. This effect was successfully used to maintain the selectivity of Ca^2+^ over Na^+^ with an increasing current density.

### 4.3. Concentration and Composition

The solution composition plays a crucial role in how the current is distributed among competing counter-ions. An accurate understanding of the influence of electrolyte concentrations and composition on ion selectivity is thus needed to develop electrodialysis applications with preferential removal between counter-ions. The influence of solution concentration on ion selectivity has been studied by different groups, both theoretically [194,195,196] and experimentally [162,172,197,198,199,200]. It is coherently reported that the ion selectivity decreases with increasing salinity of the feed solutions. The loss of ion selectivity with salinity has also commonly been attributed to back diffusion [184] and the loss of the charge-exclusion ability of the membrane at high concentrations due to the charge screening [181,194]. As a result of their high affinity, multivalent ions can be attracted to the membrane’s fixed charges, thereby neutralizing the membrane matrix and leading to increased selectivity for monovalent over multivalent ions [201]. Conversely, charge screening could increase the permeation of multivalent ions when working with monovalent selective IEMs. If the selective layer is neutralized, the electrostatic exclusion acting on multivalent ions is reduced, and multivalent ions can permeate the membrane to a greater extent. Zhu et al. [93] studied this behavior for the selective separation of Mg^2+^ and K^+^, using monovalent selective CEMs, and found that the Mg^2+^ flux increased at higher feed concentrations. Firdarous et al. [175] studied the sensitivity of mass transfer of Na^+^, Mg^2+^, and Ca^2+^ to the presence and concentration of the respective ions. The target was the selective removal of Na^+^ over Mg^2+^ and Ca^2+^ across monovalent selective CEMs. While the divalent cation transfer fluxes showed very little sensitivity to the presence of Na^+^, the presence of divalent cations retarded the flux of Na^+^. Additionally, when both Mg^2+^ and Ca^2+^ were present in the solution, a strong effect of mutual abatement of their respective fluxes was observed. Furthermore, the transfer fluxes of Mg^2+^ and Ca^2+^ showed little sensitivity to the respective ion concentrations, while the Na^+^ flux was significantly enhanced by the Na^+^ concentration in the solution [175]. Since the divalent cations have a stronger affinity to the ion exchange sites, they block the sites for Na^+^. On the other hand, the weaker repulsion energy at the membrane–solution interface and weaker affinity to the exchange groups acting on Na^+^ allow for higher mobility. The lower number of available sites for Na^+^ is compensated for by a large diffusivity, which is sensitive to the ion concentration in the solution. Galama et al. [179] reported that lower initial concentrations of Ca^2+^ and Mg^2+^, as well as K^+^, compared to Na^+^ lead to a stronger depletion of these ions in the transport layer adjacent to the membrane surface. These boundary-layer effects are reported to be more pronounced at higher applied current densities, resulting in a reduced transport of ions with a low initial concentration. Consequently, when competing counter-ions exist in different concentrations, the current density can control the selectivity between counter-ions, as proven by Zimmermann et al. [7]. The membrane selectivity decreases when the diluate concentration falls below a certain threshold [171,202], which can be attributed to the loss of ionic strength in the solution and boundary layer, causing high resistance and shifting kinetic control from the membrane matrix to the boundary layer, in a similar way as for increasing current density. Continuous operation or an alternative process design, such as multi-step electrodialysis, could be a solution to counteract this performance drop.

### 4.4. Process Design

The ion transport rates and selectivity in electrodialysis can be enhanced by optimizing the process design. Efforts to increase the ion removal rate during electrodialysis desalination include multistage electrodialysis [203,204], feed-forward voltage control of the electrodialysis unit [205], and dynamic current density [206]. Multistage electrodialysis can be adopted to keep the ion concentration gradient across the membrane below a certain threshold, which can be used to increase ion selectivity in multi-ionic mixtures. Time-variant voltage control can raise the average ratio of applied current to limiting current density and, consequently, increase the rate of ion transfer. An alternative electrodialysis stack configuration referred to as selectrodialysis was proposed by Zhang et al. [207]. The process aims to fractionate counter-ions of different valences while simultaneously desalinating the feed solution. A unit cell in selectrodialysis consists of cell triplets rather than cell pairs. A cell triplet is formed by stacking a non-selective AEM, a non-selective CEM, and a monovalent selective CEM for the fractionation of cations. During the selectrodialysis, three major effects occur: (1) the feed water between the AEM and CEM gets desalinated, (2) the multivalent ion concentration in the product between the CEM and the monovalent CEM increases, and (3) the brine between the monovalent CEM and AEM becomes more concentrated in ions. In this way, not only is the feed solution desalinated but also the product stream fractionated, meaning that the multivalent ions are collected as a separate product. Ghyselbrecht et al. [208] used this concept to recover Mg^2+^ from seawater. In another study, Zn^2+^ and Cu^2+^ were recovered from acidic metallurgical process streams, where arsenic (mainly present as anionic species H_2_AsO_4_^−^) and Na^+^ were collected in the waste stream [209]. This last example insinuates the importance of considering the speciation of different cations when treating multi-ionic mixtures, as it varies with process parameters such as pH, temperature, and concentration.

### 4.5. pH and Temperature 

Cifuentes et al. [210] studied the electrodialysis of H_2_SO_4_-CuSO_4_ electrolytes with metallic impurities in the form of As and Sb. Both As and Sb could be present as neutral complexes, cationic (i.e., H_4_AsO_3_^+^) or anionic (i.e., H_2_AsO_4_^−^) species, depending on the oxidation state of the dissolved species and the solution pH and temperature. For As and Cu^2+^, the concentration of dissolved species was highly dependent on temperature, acidity, and concentration. An increase in the solution temperature from 22 °C to 44 °C caused a nearly 40% increase in the Cu^2+^ transport rate, which was ascribed to the enhanced dissociation of Cu^2+^ with temperature and the increasing solution conductivity that correlates positively with ion mobility. However, the Cu^2+^/H^+^ ratio was expected to decrease with temperature due to a more pronounced decrease in the association of H^+^ than Cu^2+^. The solution temperature is further negatively correlated with the viscosity, which influences the mass transfer by diffusion. Nie et al. [174] compared the selectivity between Li^+^ and Mg^2+^ at 15 °C and 30 °C and found that selectivity for Li^+^ over Mg^2+^ was higher at the lower temperature value. The enhanced conductivity with increasing temperature benefits multivalent ions due to their greater charge density compared to monovalent ions, whereas the increasing significance of diffusivity at lower temperatures favors the relative transport of monovalent over multivalent ions.

Evidently, varying the pH and temperature can be a strategy to manipulate counter-ion selectivity by controlling the degree of complexation/dissociation and the relative transport rates of different species in a multi-ionic mixture. However, studies on the influence of temperature and pH on the selective separation between counter-ions are scarce and, hence, present a possible target for future research. 

### 4.6. Solvent 

A few studies were carried out on the effect of solvent on ion selectivity [175,211,212,213]. Kameche et al. [213] compared the relative transport rates between protons and Li^+^, Na^+^, K^+^, and Cs^+^ through a CEM when using water, methanol, N-methyl-formamide, or acetonitrile. It was found that organic solvents increased the transport rates of the alkali metal cations while decreasing the protonic transport rate. Rottiers et al. [173] studied the effect of different solvents on competitive transport between protons, Na^+^, and Ca^2+^. Pure water as a solvent was compared to water/methanol and water/ethanol mixtures. The amount of nonpolar solvent favored the transport of Ca^2+^ over Na^+^ due to its influence on ion mobility. The conductivity (and, thus, mobility) of Ca^2+^ was higher when using nonpolar co-solvents than pure water, while the opposite was observed for Na^+^. However, ion selectivity between Na^+^ and H^+^ was enhanced when using a nonpolar solvent compared to water, as the conductivity loss in the mixed solvent was higher for protons than Na^+^. The influence of ethanol was larger than that of methanol due to its higher non-polarity. Using different solvents for achieving increased mobility of the target species and/or restricting the conductivity of the competing species is a promising, yet little investigated, approach for manipulating counter-ion selectivity in electrodialysis. 

## 5. Conclusions

Basic applications of CEMs in the electrodialysis process include the recovery and enrichment of desired metal ions and removal of unwanted species from process streams. Standard CEMs can achieve this task to some extent but provide a limited separation ability between counter-ions. Fortunately, advances in monovalent selective CEM development and new findings in electrodialysis process design have made it possible to separate metal cations of different or equal valences through CEMs.

In the presence of competing metal ions, different strategies have been found to be effective for improving ion selectivity. Depending on the ionic characteristics, choosing a suitable membrane preparation method is of pivotal importance to achieve the intended selectivity. Surface modification by an opposite-charged polyelectrolyte layer can offer outstanding selective separation properties due to creating affinity differences between competing metal ions. However, the addition of several charged layers on a membrane surface tends to increase the surface electrical resistance and instability of the modified layers. In the case of the active layer detachment from the support membrane, multilayers can be constructed by electric-pulse and alternating current deposition technology or surface chemical modification. 

Regulating the CEM matrices can also be an effective strategy to enhance ion selectivity. The increased polarity difference between hydrophilic and hydrophobic segments within a CEM matrix renders a more obvious nano-phase separation with an ordered morphology, and the precise control of ion channels within the nanoscale can effectively improve the transport and selectivity of cations.

Adding and functionalizing inorganic particles or fillers can also improve ion selectivity between monovalent and divalent cations. Inorganic phases embedded within a polymeric matrix might have different components, such as metal oxides, MOF, graphene oxide, and carbon nanotubes, through which the selective mechanisms are mainly governed by the electrostatic repulsive force interaction and pore-size sieving effect. 

The flow rate significantly influences counter-ion selectivity in both common CEMs and monovalent selective CEMs. Increasing flow rate promotes the transport of multivalent ions over monovalent ions in common CEMs, while the opposite occurs in monovalent selective CEMs. Increasing the ion selectivity often involves reducing the boundary layer thickness by enhanced mixing. However, the boundary layer can also be exploited to selectively transport one cation over another, particularly when the competing cations have different concentrations. A lower concentration of a species relative to another leads to more complete depletion of that species in the boundary layer. Understanding the current distribution among competing counter-ions is crucial to determine when a species’ mass transport becomes diffusion-limited. The differential mass transport control based on ion concentration and applied current is an important tool for controlling ion selectivity in conjunction with membrane characteristics.

## 6. Outlook

Tailor-made CEMs and process optimization for separating metal ions offer promising performances in electrodialysis applications. However, the selective separation of certain metals remains at moderate values. More research is needed to develop new robust CEMs with increased ion selectivity, specifically between metal ions of the same valency. Moreover, the practical application of CEMs in electrodialysis for the selective recovery of precious and noble metals is scarce and requires urgent attention. Furthermore, more work needs to be performed to increase long-term stability without increasing the membrane surface resistance, while maintaining a high ion selectivity. Knowledge gaps should be clarified with regard to the effects of temperature, pH, and solvent in electrodialysis with counter-ion selectivity. Additionally, more research is needed on the feasibility of separating cations with the same charge sign and removing trace concentrations of target cations in multi-ionic mixtures, since these cases prevail in the industrial processes where electrodialysis with cations selectivity is of interest.

## Figures and Tables

**Table 1:** Ionic characteristics of metal cations.

Cation	Ionic Radius (pm) [36]	Hydrated Radius (pm) [36]	Hydration Free Energy (kJ⋅mol^−1^) [38]	Charge Density (C⋅mm^−3^) [27]
$Li^{+}$	60	382	−475	98
$Na^{+}$	95	358	−365	24
$K^{+}$	133	331	−295	11
$Cs^{+}$	181	329	−250	6
$Mg^{2+}$	65	428	−1830	120
$Ca^{2+}$	99	412	−1505	52
$Cu^{2+}$	72	419	−2010	116
$Ni^{2+}$	70	404	−1980	134
$Co^{2+}$	72	423	−1915	108
$Fe^{2+}$	92	428	−1840	98
$Fe^{3+}$	60	457	−4265	232
$Cr^{3+}$	64	461	−4010	261
$Zn^{2+}$	74	430	−1955	112
$Al^{3+}$	50	475	−4525	364

**Table 2:** Reported ion selectivity of LbL-modified CEMs in electrodialysis.

Commercial Membrane	(Polyelectrolyte Pair)_n_	Feeding Solution (Diluted Cell)	Current Density (mA⋅cm^−2^)	Ion Selectivity			Ref.
				Ion Pair	Value	Time	
Fujifilm CEM	(PAH/PSS)_6_PAH	0.025 M NaCl 0.01 M MgCl_2_	-	$Na^{+}/Mg^{2+}$	7.8	-	[87]
CMX CEM	(PEI/PSS)_5_PEI	0.05 M NaCl 0.05 M CaCl_2_	15	$Na^{+}/Ca^{2+}$	1.35	6 h	[89]
Nafion 115 CEM	(PAH/PSS)_5_PAH	0.01 M LiNO_3_	0.63	$Li^{+}/Co^{2+}$	>1000	90 min	[90]
		0.01 M Co(NO_3_)_2_					
		0.01 M K(OAc)		$K^{+}/La^{2+}$	>93		
		0.01 M La(OAc)_3_					
Whatman Alumina membrane	(PSS/PAH)_5_	0.01 M KCl	7.7	$K^{+}/Mg^{2+}$	>390	20 min	[91]
		0.01 M MgCl_2_			4.3	1.5 h	
		0.01 M KNO_3_					
		0.01 M Mg(NO_3_)_2_			>340	-	
Nafion 115 CEM	(PAH/PSS)_5_PAH	0.01 M KNO_3_ 0.01 M Mg(NO_3_)_2_	1.27	$K^{+}/Mg^{2+}$	>1000	30 min	[92]
Fujifilm CEM	(PDADMAC/PSS)_5_ PDADMAC	0.01 M KNO_3_	6.3	$K^{+}/Mg^{2+}$	>1000	2 h	[93]
		0.01 M Mg(NO_3_)_2_					
		0.01 M LiNO_3_		$Li^{+}/Co^{2+}Co$	>1000		
		0.01 M Co(NO_3_)_2_					
Nafion 115	(PAH/PSS)_5_PAH	0.01 M KCl 0.01 M MgCl_2_	3.42	$K^{+}/Mg^{2+}$	32	2 h	[94]
Nafion 115 CEM	(PAH/PSS)_5_PAH	0.01 M KNO_3_ 0.01 M Mg(NO_3_)_2_ 0.01 M LiNO_3_	0.8	$K^{+}/Mg^{2+}\;Li^{+}/Mg^{2+}$	>100	6 h	[95]
Nafion 115	(PAH/PSS)_5_PAH	0.01 M KNO_3_ 0.01 M LiNO_3_	0.64	$K^{+}/Li^{+}$	7	-	[96]

**Table 3:** Overview of studies on separation of monovalent and divalent ions with electrodialysis.

Commercial Membrane	U (V)	Current Density (A⋅m^−2^)	Stack Size	A (m^2^)	*v_c_*	*v_d_*	*v_r_*	*d_s_*	Rinse Solution	S (t) (%)	Ion Selectivity		Ref
					(L⋅m^−1^)	(L⋅m^−1^)	(L⋅m^−1^)	(mm)			Ion Pair	Value	
Neosepta	-	div.	10	0.43	95	95	95	0.5	0.2 M Na_2_SO_4_	-	$Na^{+}/Ca^{2+}\;Na^{+}/Mg^{2+}$	3.6–4.6 5.7–8.7	[171]
Selemion CSO/ASV	-	40 and 100	5	0.0945	40	40	40	-	Na_2_SO_4_	27 and 25 56 and 54	$Na^{+}/Ca^{2+}\;Na^{+}/Mg^{2+}$	-	[172]
Neosepta CIMS/ACS	-	40 and 100	5	0.0945	40	40	40	-	Na_2_SO_4_	40 and 35 66 and 63	$Na^{+}/Ca^{2+}\;Na^{+}/Mg^{2+}$	-	[172]
Neosepta CMS	-	150–500	2	-	-	-	-	-	0.5 M H_2_KNO_3_S	-	$Na^{+}/Ca^{2+}$	1–8	[173]
Selemion CSO/ASA	-	5.9–13.8	20	0.0507	-	6000–12,000	-	0.75	NaCl (cat.) Na_2_SO_4_ (an.)	20.2–0.33	$Li^{+}/Mg^{2+}$	-	[174]
Neosepta ACS/CMX-S	-	55–323	16	0.01	57.6	57.6	120	-	0.1 M Na_2_SO_4_	*-*	*various*	-	[63]
Neosepta ACS/CMX-S	30	-	20	0.138	200	200	200	-	0.17 M NaCl	-	$Na^{+}/Ca^{2+}/Mg^{2+}$	-	[175]
Modified Nafion 324	-	150	10	0.02	20	20	20	-	0.25 M Na_2_SO_4_	0.02–0.84 0.33–0.89	$Na^{+}/Cr^{3+}$	-	[105]
Neosepta CMX/AMX CMS/ACS	-	20–200	14	0.1456	0.9	0.9	7.2	-	0.5 M NaCl	0.21–0.75	$Na^{+}/Mg^{2+}$	-	[176]
PC-SA/SK PC-MVA/SK	-	3.125–15.625	5	0.032	30	30	150	0.5	0.1 M H_2_SO_4_	−0.04–0.20 0.01–0.07		-	[177]
PC-SA/SK PC-MVA/SK	-	3.125–15.625	5	0.032	30	30	150	0.5	0.1 M H_2_SO_4_	−0.23–0.28 −0.04–0.08		-	[177]
PC-SA/SK PC-MVA/SK	-	3.125–15.625	5	0.032	30	30	150	0.5	0.1 M H_2_SO_4_	-		-	[177]
PC-SA/SK PC-MVA/SK	-	3.125–15.625	5	0.032	30	30	150	0.5	0.1 M H_2_SO_4_	-		-	
CIMS TWDDC1 TFC-CIMS	-	50–500	1	0.002	20	40	10	20	0.255 M Na_2_SO_4_	-	$Na^{+}/Mg^{2+}$	50–100	[178]
Selemion CMV/AMV	-	-	10	0.0563	12, 24, 26, 48	12, 24, 26, 48	78	0.408	each 10 mM KNO_3_ and Ca(CH_3_COO)_2_	-	$K^{+}/Ca^{2+}$	-	[22]
Neosepta CMX/AMX	-	10–300	9	0.1872	0.9	0.9	6	0.5	0.5 M NaCl	-	$Na^{+}/Mg^{2+}/Ca^{2+}$	10–300	[179]
Neosepta CMX/AMX	7.26	-	10	0.1	96	96	-	-	-	-		-	[180]
Neosepta CMX/AMX	-	37.5–275	10	0.08	80	80	180	-	0.3 M Na_2_SO_4_	-	$Na^{+}/Mg^{2+}$	37.5–275	[181]
Neosepta CMX/AMX	5, 10	-	10	0.1	30, 96	30, 96	30, 96	-	-	-	$Na^{+}/K^{+}/Mg^{2+}/Ca^{2+}$	-	[182]
Neosepta CMX/CMS/AMX	-	62–143	1	0.0011	60	60	60	-	0.5 M Na_2_SO_4_	-	$Na^{+}/Ca^{2+}$	0.46–2.68	[183]
Neosepta CMX-fg/AMX-fg	10, 15	-	10	0.0064	0.5	0.5	-	-	-	-	$Na^{+}/K^{+}$	-	[184]

## Data Availability

Not applicable.
